# Dimensionality Control of Inorganic and Hybrid Perovskite Nanocrystals by Reaction Temperature: From No‐Confinement to 3D and 1D Quantum Confinement

**DOI:** 10.1002/anie.202109308

**Published:** 2021-11-15

**Authors:** Clara Otero‐Martínez, Daniel García‐Lojo, Isabel Pastoriza‐Santos, Jorge Pérez‐Juste, Lakshminarayana Polavarapu

**Affiliations:** ^1^ Department of Physical Chemistry CINBIO Universidade de Vigo, Materials Chemistry and Physics Group Campus Universitario As Lagoas, Marcosende 36310 Vigo Spain; ^2^ Department of Physical Chemistry CINBIO Universidade de Vigo Campus Universitario As Lagoas, Marcosende 36310 Vigo Spain; ^3^ Galicia Sur Health Research Institute (IIS Galicia Sur) SERGAS-UVIGO Vigo Spain

**Keywords:** CsPbX_3_, FAPbX_3_, nanocubes, nanoplatelets, quantum confinement

## Abstract

This work focuses on the systematic investigation of the shape, size, and composition‐controlled synthesis of perovskite nanocrystals (NCs) under inert gas‐free conditions and using pre‐synthesized precursor stock solutions. In the case of CsPbBr_3_ NCs, we find that the lowering of reaction temperature from ∼175 to 100 °C initially leads to a change of morphology from bulk‐like 3D nanocubes to 0D nanocubes with 3D‐quantum confinement, while at temperatures below 100 °C the reaction yields 2D nanoplatelets (NPls) with 1D‐quantum confinement. However, to our surprise, at higher temperatures (∼215 °C), the reaction yields CsPbBr_3_ hexapod NCs, which have been rarely reported. The synthesis is scalable, and their halide composition is tunable by simply using different combinations of precursor solutions. The versatility of the synthesis is demonstrated by applying it to relatively less explored shape‐controlled synthesis of FAPbBr_3_ NCs. Despite the synthesis carried out in the air, both the inorganic and hybrid perovskite NCs exhibit nearly‐narrow emission without applying any size‐selective separation, and it is precisely tunable by controlling the reaction temperature.

## Graphical abstract

The morphology of both inorganic and hybrid halide perovskite nanocrystals is tunable from 3D nanocubes with no‐confinement to 0D nanocubes and 2D nanoplatelets with 3D and 1D‐quantum confinement, respectively by a decrease of the reaction temperature in the hot‐plate approach under inert gas‐free conditions. The synthesis is scalable, and their halide composition is tunable by simply using different combinations of precursor stock solutions.

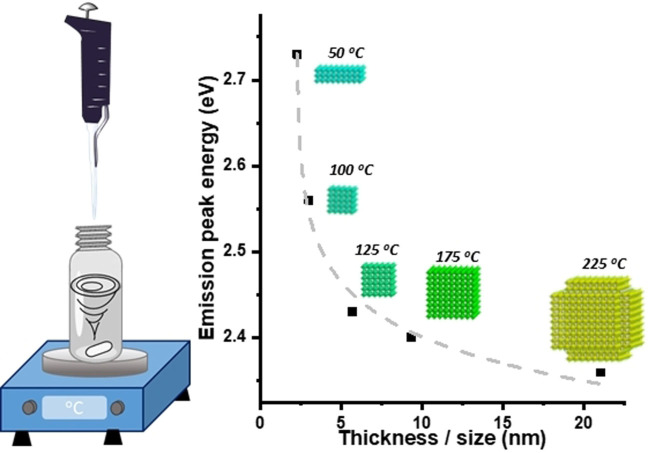

## Introduction

Colloidal lead halide perovskite nanocrystals (LHP NCs) have recently emerged as a new class of light‐emitting semiconductors having a great potential for a wide range of potential applications owing to their interesting optical and electronic properties.^[1]^ In particular, the high photoluminescence quantum yield and the tunable emission across the visible spectrum of light make them attractive light sources for a wide range of applications such as light‐emitting devices (LEDs), displays, and lasers.[^1f^, ^1k^, ^2^] The optical properties of LHP NCs are easily tunable by their halide (Cl, Br, and I) composition as well as by their dimensions.[^1a^, ^1d^, ^3^] For instance, 2D nanoplatelets (NPls) and 0D nanocubes of LHPs exhibit strong quantum confinement effects and the optical properties are tunable by their thickness and size, respectively.[^1a^, ^1d^, ^3a^, ^4^] The 3D, 2D, and 0D refer to the free movement of electrons in 3, 2, and 0 dimensions, respectively, in other words, the electrons are confined in 0, 1 and 3 dimensions, respectively. For instance, 3D nanocubes exhibit no quantum confinement in 3 dimensions and show bulk‐like optical properties. On the other hand, 0D nanocubes exhibit 3D‐quantum confinement, while the 2D NPls exhibit 1D‐quantum confinement. The another important feature of LHP NCs is that they exhibit higher stability compared to bulk thin‐film perovskites due to their surface protection by capping ligands. This has opened doors for the fabrication of relatively stable perovskite solar cells using LHP NCs.^[5]^ The demand for LHP NCs in many potential technological applications has motivated chemists toward the development of facile synthesis methods.[^1b^, ^1i^, ^3c^, ^4a^, ^6^]

**Figure 1:**
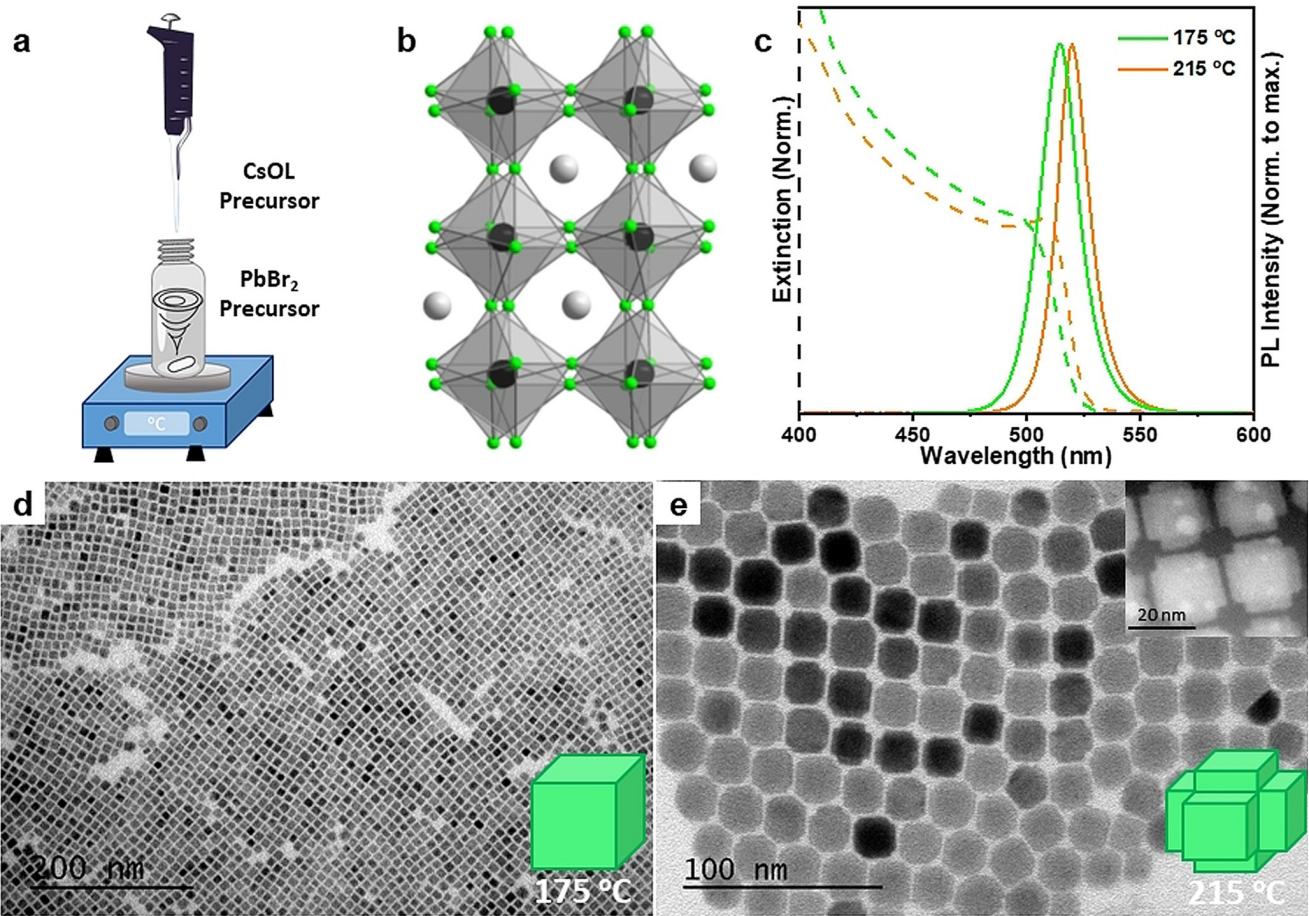
(a) Schematic illustration showing the hot‐plate synthesis of CsPbBr_3_ NCs by addition of Cs‐oleate into PbBr_2_ precursor stock solution at different temperatures. (b) General orthorhombic crystal structure of CsPbBr_3_ NCs. (c) Extinction (dashed line) and PL (solid line) spectra of CsPbBr_3_ NC colloidal dispersions synthesized at 175 °C and 215 °C reaction temperatures. (d,e) TEM images of the CsPbBr_3_ NCs prepared at two different reaction temperatures. The insets (bottom, right) in (d and e) is the schematic illustration of the shape of the NCs in respective TEM images. The inset (top, right) in (e) is a HAAD‐STEM image of CsPbBr_3_ hexapod NCs obtained at 215 °C (see Figure S2 for large area HAAD‐STEM images).

Over the years, various strategies such as hot‐injection,^[1b]^ ligand‐assisted reprecipitation (LARP),[^1h^, ^6c^] ultrasonication,[^3c^, ^7^] solvothermal,^[8]^ microwave,^[9]^ and ball milling^[10]^ have been exploited for the shape‐controlled synthesis of LHP NCs. Among all, LARP and hot‐injection synthesis are the most widely used approaches for both organic‐inorganic hybrid and all‐inorganic perovskite NCs. Although the LARP approach is well established for monodisperse nanocubes,^[6a]^ it often yields polydisperse NPls and nanowires.^[11]^ On the other hand, the hot‐injection synthesis offers better shape control and size distribution of LHP NCs.[^1a^, ^1b^, ^4a^, ^12^] This approach is generally based on the injection of a pre‐synthesized monovalent cation precursor (for example, cesium‐ oleate for all‐inorganic LHP NCs) into a freshly prepared lead halide precursor solution at high temperature and under inert conditions.^[1b]^ In fact, the synthesis of monovalent cation precursors has also been generally carried out under an inert atmosphere. Although hot‐injection synthesis has proven to be an excellent approach for the synthesis of LHP NCs, it is laborious and requires inert conditions. The first question is: can we synthesize high‐quality colloidal perovskite NCs with shape and size control under inert gas‐free conditions? On the other hand, the LHP NCs prepared by hot‐injection synthesis exhibit blue‐shifted emission with a decrease in reaction temperature. This was attributed differently (either to decrease in the size of nanocubes or to the formation of nanoplatelets) in different reports.[^1b^, ^4a^] Therefore, the morphology and the PL emission of corresponding NCs obtained at different temperatures is not fully understood. To address these questions, herein we systematically investigated the synthesis of colloidal LHP NCs in the air using pre‐synthesized precursor stock solutions at different reaction temperatures. We find that the decrease of reaction temperature from ≈175 to 100 °C leads change in morphology from bulk‐like 3D nanocubs to strongly confined 0D nanocubes, while at temperatures below 100 °C 2D NPls with are obtained. However, at very higher temperatures (>200 °C), the reaction yields hexapod NCs in the case of CsPbBr_3_. Such hexapods NCs were previously reported through a multi‐step process. The optical properties of the resultant LHP NCs can be precisely tailored through the control of the reaction temperature. In addition, the halide composition of the NCs and thus their emission color is varied by simply using different ratios of corresponding pre‐synthesized precursor solutions. The synthesis is also applicable to organic‐inorganic hybrid perovskite NCs. For instance, we have demonstrated the synthesis of FAPbBr_3_ nanocubes of different sizes and nanoplatelets of different thicknesses by controlling the reaction temperature. The prepared FAPbBr_3_ NCs exhibit narrow emission with a single PL peak suggesting the shape purify of the prepared NCs. The synthesis of perovskite NCs on a hot‐plate is illustrated in Figure 1 a. This method is based on the simple addition of a Cs‐oleate solution to a PbX_2_ (X=Cl, Br, or I) precursor solution at a certain temperature. The stock solutions of precursors are prepared by dissolving their respective salts in octadecene with the help of ligands (oleylamine and oleic acid) at 125 °C under atmospheric conditions (see the experimental section in the Supporting Information for detailed description). The precursor stock solutions are stable for more than 6 months and can be readily used to prepare perovskite NCs in large quantities. This hot‐plate approach is inspired by the well‐studied hot‐injection synthesis of perovskite NCs,^[1b]^ however, the pre‐synthesized stock solutions and the synthesis under atmospheric conditions make this approach faster, user‐friendly, scalable, and more practical for device applications.

## Results and Discussion

We first studied the influence of the temperature in the synthesis of CsPbBr_3_ NCs. Thus, a pre‐heated Cs‐oleate precursor solution was injected into a PbBr_2_ precursor solution at 175 °C or 215 °C under vigorous stirring (see Figure 1 a and the experimental section in Supporting Information for more details). In both experiments, the colorless reaction medium immediately turns yellow and exhibits intense green emission under UV light illumination, indicating the formation of CsPbBr_3_ NCs. Although initial studies claimed that CsPbBr_3_ NCs exhibit cubic crystal structure, recently it has been widely accepted that they crystalize in the orthorhombic phase, as illustrated in Figure 1 b.[^6b^, ^6d^] As depicted in Figure 1 c, the purified colloidal solutions in hexane exhibit absorption onsets at ∼510 and 515 nm with narrow emission and symmetrical PL peaks at ∼515 and 520 nm for samples synthesized at 175 °C and 215 °C, respectively. The absorption and emission peaks are typical for 3D CsPbBr_3_ NCs that exhibit bulk like optical properties. The shape and size distribution of the CsPbBr_3_ colloidal NCs is studied by transmission electron microscopy (TEM). The TEM images (Figure 1 d and 1e) shows that the CsPbBr_3_ NCs prepared at 175 °C are nearly‐monodisperse nanocubes with an average edge length of 9.3±1.1 nm, while the NCs obtained at 215 °C exhibit hexapod morphology with size ∼20 nm (also see Figure S1 for HAAD‐STEM images of hexapods). In both the cases, the size of CsPbBr_3_ NCs is higher than their exciton Bohr radius (∼7 nm) and therefore they exhibit bulk‐like optical properties.^[1b]^ The photoluminescence quantum yields (PLQYs) for nanocubes and hexapods were measured to be 95 % and 5 %, respectively (see Table S2 in SI). The low PLQY of hexapods could be due to the presence of defects that needs further in‐depth spectroscopic investigation. However, the monodispersity of the CsPbBr_3_ nanocubes with a standard deviation of ∼10 % and the PLQY of ∼95 % obtained by this hot‐plate approach seems to be similar to those obtained by classical hot‐injection synthesis under degassed and inert conditions.[^1b^, ^4a^, ^6b^, ^13^] The crystal phase of the obtained nanocubes and hexapods were characterized by powder X‐Ray diffraction (Figure S3), and the pattern resembles the orthorhombic phase according to the literature.^[14]^ The scalability of this approach is demonstrated by increasing the volume of the two precursors in the reaction medium by 25 times to obtain 160 mL of CsPbBr_3_ NC colloidal solution in a single run, with the same concentration as in small‐scale synthesis. The CsPbBr_3_ NCs thus obtained exhibit the optical features and morphology (nanocubes of ∼9.3 nm) similar to those prepared in small‐scale synthesis (see Figure S2). This makes the hot‐plate approach promising for obtaining perovskite NCs for industrial‐scale device applications in the future.

**Figure 2:**
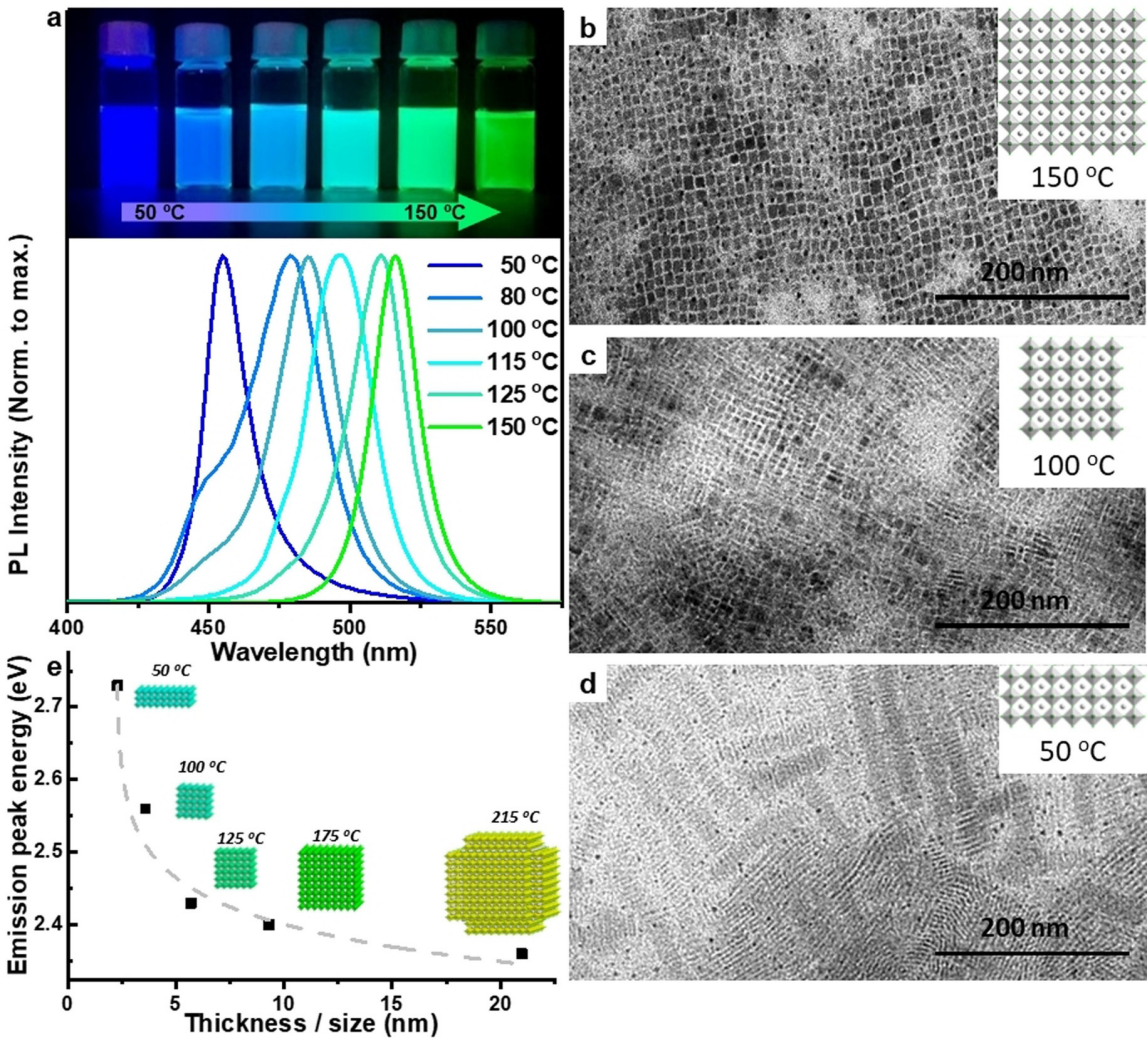
(a) Normalized photoluminescent spectra of the CsPbBr_3_ colloidal solutions synthesized at different reaction temperatures. The inset (top) is the photograph of the corresponding colloidal dispersions under UV light illumination. (b‐d) TEM images of the CsPbBr_3_ NCs obtained at 150 °C (b), 100 °C (c) and 50 °C (d). The insets are the schematics of the shape and size of NCs in terms of the number of octahedral monolayers. (e) Emission peak energy versus nanoplatelets thickness and cubic size. Dashed line is added as a guide the eye. Insets are the corresponding schematic representations of the structures obtained and the reaction temperature employed.

On the other hand, the hexapods obtained at 215 °C also exhibit excellent monodispersity with an increase in size compared to nanocubes. The increase of size clearly reflects in the red‐shifted absorption and emission spectra of hexapods. In fact, the formation of hexapods at higher temperatures has come as surprise to us. Such multi‐faceted hexapod perovskite NCs have been very rarely reported in the literature.[^1a^, ^14^] For instance, Peng et al.^[14]^ reported the synthesis of such hexapod NCs by hot injection synthesis using pre‐synthesized and purified CsPbBr_3_ nanocubes as seeds and oleylamine‐hydrohalic acid complex. It was claimed the seeded growth of hexapods occurs through intermediate polyhedron‐shaped NCs under halide‐deficient conditions. In the present work, similar hexapod NCs are obtained in a one‐pot hot‐plate approach. However, the growth mechanism is currently unclear. The results clearly demonstrate the importance of reaction temperature on the morphology of the resulting perovskite NCs. Therefore, we have systematically investigated the influence of reaction temperature on the morphology and optical properties of the resultant CsPbBr_3_ perovskite NCs by performing the synthesis under different temperatures ranging from 150 to 50 °C (Figure 2). Figure 2 a shows that the emission color of the resulting colloidal dispersions shows a clear shift from green to deep blue and the corresponding PL spectra show a gradual shift in the photoluminescence from 516 nm to 455 nm with decreasing the reaction temperature (Figure 2 a). Previously, Alivasatos and co‐workers^[4a]^ reported such blue‐shifted peaks of CsPbBr_3_ NCs for NPls of different thicknesses, while Kovalenko and co‐workers^[1b]^ reported for CsPbBr_3_ nanocubes of different edge lengths. In both reports, the NCs were prepared by classical hot‐injection synthesis at different reaction temperatures.

Therefore, herein, we carried out TEM analysis of our samples to understand the blue‐shifted emission in this work (Figure 2 b–d & S4‐S6 (Supporting Information)). The PL emission peak, PLQY, and morphology of NCs obtained at different reaction temperatures are summarized in Table S2 in the supporting information. Interestingly, we find that that the morphology NCs obtained in the temperature range of 175‐125 °C remains cubic with a decrease of their average edge length from ≈10 nm to 5.7 nm, and they all exhibit over 90 % PLQY (see Figures 1 d, 2b & S4 for 175, 150, and 125 °C, respectively, and Table S2). However, further decreasing the reaction temperature to 100 °C leads to the formation of NPls together with nanocubes (Figure 2 c, and see Figure S5 for large‐area images). Based on the TEM images, the estimated thickness of the NPls and the size of nanocubes obtained are ∼1.9 nm (3‐ monolayer‐thick) and ∼3.7 nm, respectively (Figure S5). The size of the nanocubes is well‐below the exciton Bohr radius (∼7 nm), thus they exhibit 3D‐quantum confinement.^[1b]^ Interestingly, at 50 °C monodisperse 3‐monolayer‐thick NPls with 1D‐quantum confined are obtained (Figures 2 d & S6), and they exhibit orthorhombic crystal phase similar to nanocubes (Figure S3).^[15]^ These results suggest that, in our case, the initial blue shift in the PL peaks from ∼515 nm to ∼470 nm is due to the decrease of the size of nanocubes, while the further blue shift down to 455 nm is caused by the formation of 3‐monolayer‐thick NPls. The PL spectra of the NPls show one main peak with a small shoulder peak and this indicates that the colloidal solution contains nearly‐monodisperse NPls of single thickness with only a very little contribution of other thicknesses. However, the NPls exhibit low PLQY (5 %) compared to that of nanocubes (95 %). The low PLQY of NPls is due to large number of defects caused by high surface area, and is well known from literature that they require post‐synthetic surface passivation to achieve high PLQY.^[15]^ Furthermore, we demonstrate that the synthesis of CsPbBr_3_ NPls also scalable similar to that of CsPbBr_3_ nanocubes by increasing the volume of the precursors in the reaction medium by 20 times to obtain a final volume of 130 mL with the same thickness of NPls obtained in small‐scale synthesis (See the characterization of NPls in Figures S6 for small‐scale and S7 for large‐scale). However, we find that the strongly quantum confined perovskite nanocubes and NPls exhibit relatively poor long‐term stability compared to 3D nanocubes, as it was previously reported.^[3b]^

Overall, regardless of the morphology, it is clear that the PL peak position of CsPbBr_3_ NCs can be precisely tuned across from blue to green by controlling the reaction temperature. Figure 2 e illustrates the PL energy of the NCs obtained at different temperatures vs. their size/thickness. One can clearly see a nonlinear increase in the energy of the PL peak as the size/thickness gets smaller. The increase in the PL energy is due to the decrees of the size of nanocubes, while the nonlinear increase caused by the formation of NPls at reaction temperatures below 125 °C. Therefore, one should carefully assign the blue shifted emission of perovskite NCs either to 0D nanocubes or 2D NPls. In addition, we demonstrate that this hot‐plate approach is also applicable to tune the optical properties of CsPbI_3_ NCs by varying the reaction temperature (Figure S8 in the Supporting Information). However, the PL spectra of the NCs obtained at lower reaction temperatures (≤80 °C) are broad due to the presence of NPls of different thicknesses.

**Figure 3:**
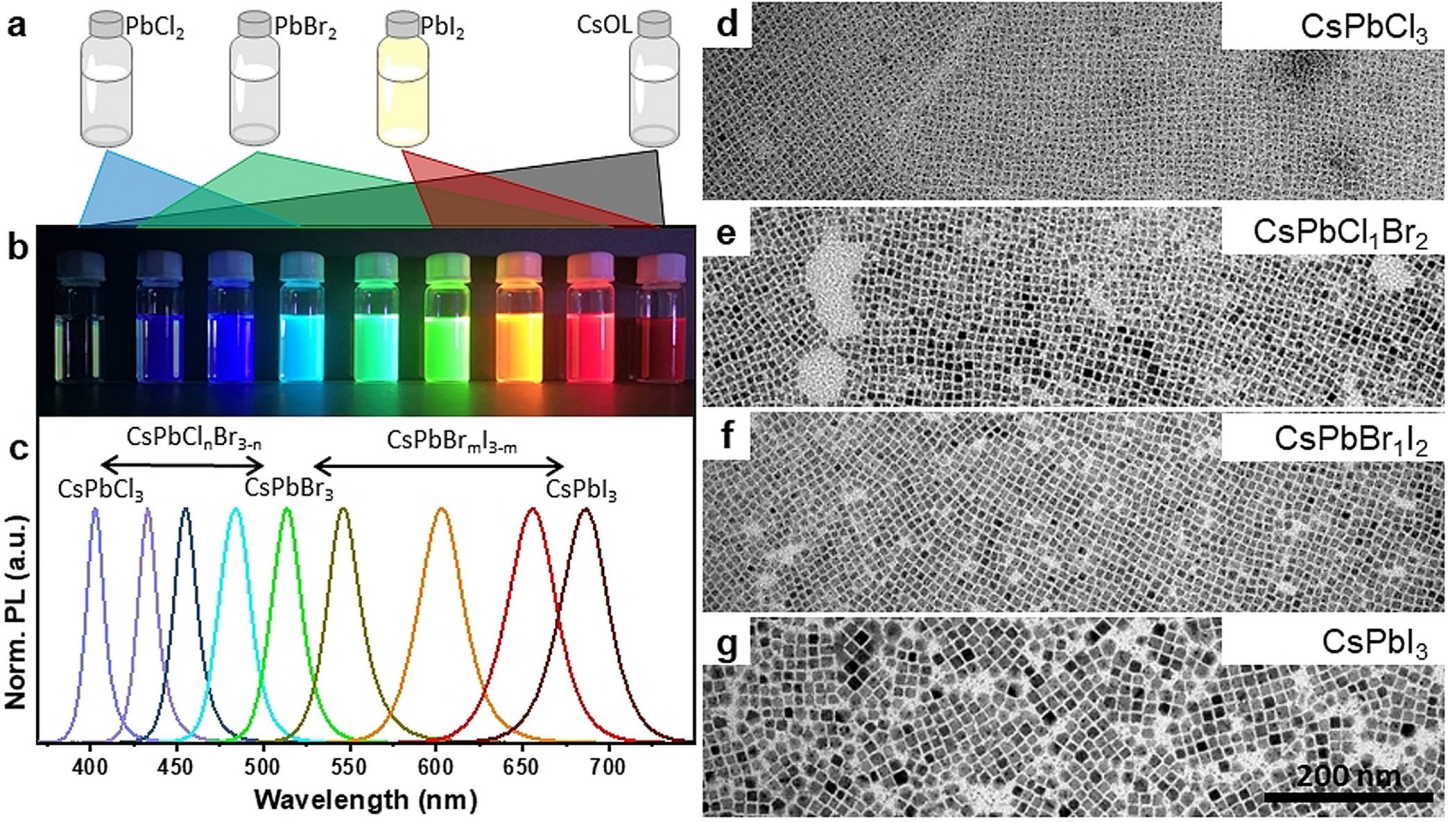
(a) Schematic illustration of the synthesis of different mixed halide CsPbX_3_ perovskite NCs using different combinations of pre‐synthesized Cs‐oleate, PbCl_2_, PbBr_2_, and PbI_2_ precursor stock solutions. (b) Photograph of the mixed halide CsPbX_3_ colloidal dispersions under UV light (395 nm). (c) Corresponding PL spectra of the colloidal dispersions. (d‐g) TEM images of CsPbCl_3_ (d), CsPbCl_1_Br_2_ (e), CsPbBr_1_I_2_ (f) and CsPbI_3_ (g) NCs. The scale bar is the same for all TEM images.

Furthermore, we demonstrate that this approach is generally applicable to the synthesis of halide perovskite NCs of any halide composition. Figure 3 a, b illustrates the synthesis of CsPbX_3_ NCs (X=Cl, Br, I, or their mixtures) of different halide compositions using corresponding combinations of PbX_2_ precursor stock solutions along with Cs‐oleate. In a typical synthesis, Cs‐oleate was added into the desired halide precursor or a mixture of different halide precursors (PbCl_2_/PbBr_2_ or PbBr_2_/PbI_2_) at 175 °C on a hot‐plate followed by cooling the reaction mixture in an ice bath (see the experimental section in Supporting Information for details). The resulting colloidal dispersions of CsPbX_3_ NCs show different light emission upon UV illumination, covering the entire visible range, which indicates different halide compositions (see Figure 3 b). The corresponding PL spectra show a narrow emission with a single peak that is tunable from 400 to 690 nm depending on the halide composition (Figure 3 c & Figure S9a). Interestingly, we find that the red‐shift of the PL peak while going from Cl to Br and Br to I is nearly‐linear with respect to their precursor ratio (see Figure S9b, c in the Supporting Information). This suggests that one can obtain a colloidal solution with a required PL peak simply by adjusting the precursor ratio according to the linear fit. However, we have to take into account that the exact halide composition of the NCs is not necessarily the same as the ratio of halides in the precursor solutions. As shown in Figure 3 d–g, the TEM images of the CsPbX_3_ NCs with different halide compositions show nearly‐monodisperse nanocubes regardless of the halide composition (Figure 3 d–g). Interestingly, the average size of the nanocubes decreases from ∼11.2 nm to 5.3 nm while going from I to Cl via Br (Figure 3 d–g, see Figure S10 and S11 in the SI for large area TEM images of CsPbI_3_ and CsPbCl_3_ NCs). This is likely due to the decrease in the size of the halide ion that reduces the lattice spacing as well as the differences in their nucleation.[^3c^, ^7^]

**Figure 4:**
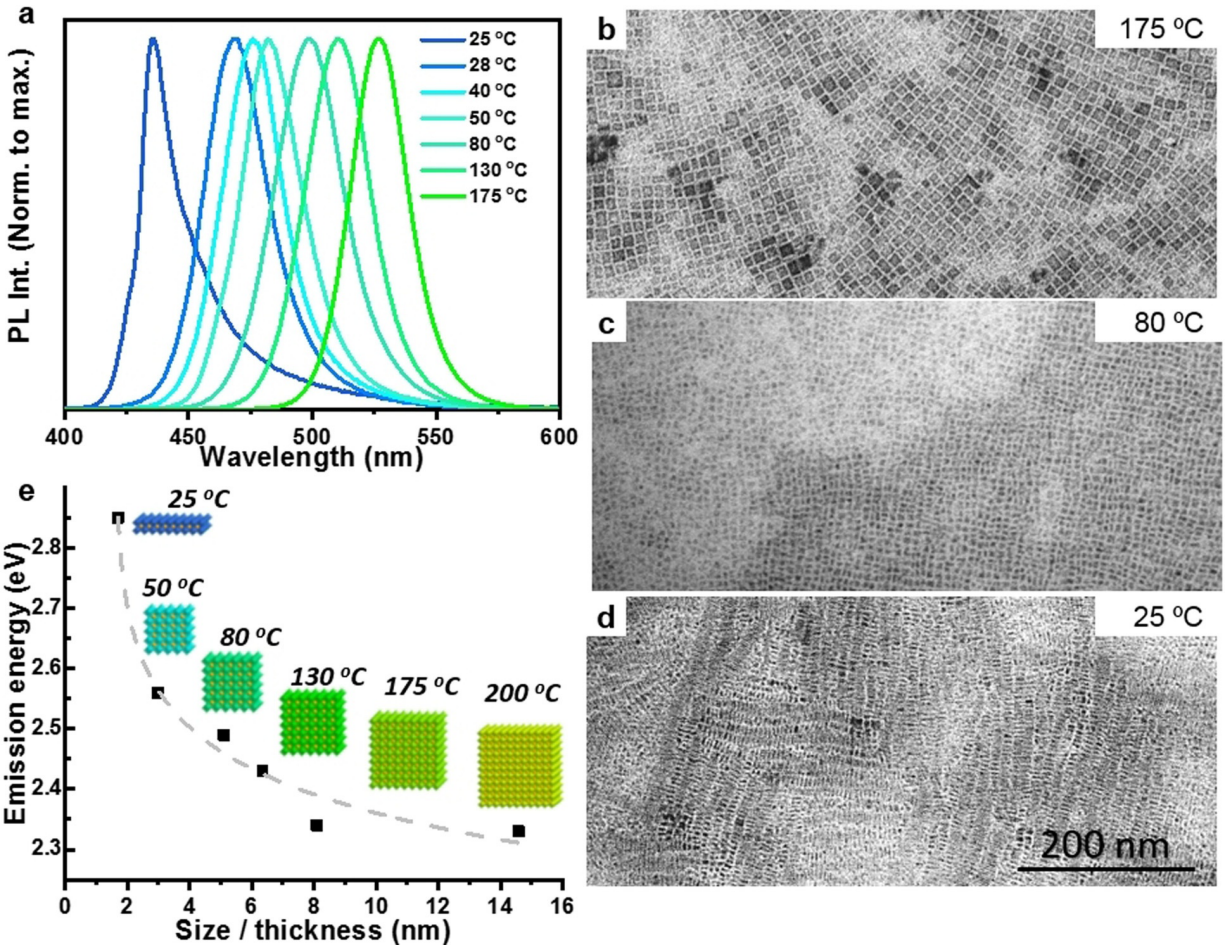
(a) Normalized photoluminescent spectra of FAPbBr_3_ NCs synthesized at different temperatures. (b‐d) TEM images of the corresponding NCs obtained obtained at 175 °C (b), 80 °C (c) and 25 °C (d). (e) Emission peak energy versus and cubic size and nanoplatelets thickness. The dashed line is a guide to the eye. Insets corresponding schematic representation of the structures obtained and the reaction temperature employed. The scale bar is the same for all TEM images.

Additionally, the versatility of this approach is verified by applying it to the synthesis of organic‐inorganic hybrid perovskites, in particular, relatively less explored formamidinium (FA) lead bromide (FAPbBr_3_) perovskite NCs by using FA‐oleate instead of Cs‐oleate (see the experimental section in the SI for details). Figure 4 a shows the PL spectra of FAPbBr_3_ NCs obtained at different reaction temperatures between 175 to 25 °C. Like in CsPbBr_3_ NCs, the PL peak of FAPbBr_3_ also blue‐shifts with decreasing the reaction temperature. Importantly, the spectra exhibit a narrow and single peak that is precisely tunable from ∼527 nm to 436 nm by varying the reaction temperature. The PL peak at 527 nm for FAPbBr_3_ NCs generally, corresponds to cubic morphology, which is also confirmed by TEM (Figure 4 b). As shown in Figure 4 b, c, the FAPbBr_3_ NCs prepared in the temperature range of 175‐80 °C exhibit cubic morphology with their average edge‐length decreasing from ∼8.1 nm to ∼5.1 nm (also see Figure S12 & S13 in the Supporting Information for large‐area TEM images). It is worth mentioning that the exciton Bohr radius of FAPbBr_3_ NCs was reported as 8 nm.^[16]^ Therefore, the decrease in edge‐length reflects the blue‐shift of PL peaks from ∼527 nm to ∼499 nm due to the strong quantum‐confinement of nanocubes obtained at 80 °C.^[4c]^ However, unlike CsPbBr_3_ nanocubes, the PLQY of nanocubes was found to be decreasing with the reduction in their size (see Table S2 in Supporting Information). The powder X‐ray diffraction pattern of the FAPbBr_3_ nanocubes (both bulk‐like and 3D‐quantum confined) resembles the cubic crystal phase reported in literature (Figure S14).^[17]^ However, further decrease of reaction temperature leads to the formation of FAPbBr_3_ 2D NPls, as shown in Figure 4 d (see Figure S15 in the Supporting Information for large area TEM image of NPls). The NPls exhibit excellent monodispersity and tend to form stacks on the TEM grid. The PL peak energy vs. the size/thickness of FAPbBr_3_ NCs obtained at different reaction temperatures, along with their morphology, is illustrated in Figure 4 e. The initial increase in PL energy of the NCs obtained by decreasing the reaction temperature from 200 to 50 °C is due to decrease of their size, suggesting the formation of strongly quantum confined 0D nanocubes. However, at room temperature strongly quantum‐confined 2D nanoplatelets with nonlinear increase in their PL energy can be seen. It is worth mentioning that 0D nanocubes and 2D NPls of FAPbBr_3_ have been less explored compared CsPbBr_3_ system. Our results demonstrate that this user‐friendly hot‐plate approach is very promising not only for inorganic perovskite NCs, but also for organic‐inorganic hybrid perovskite NCs

## Conclusion

In summary, we demonstrated a simple, scalable, and user‐friendly hot‐plate approach for the shape‐ and size‐controlled synthesis of halide perovskite nanocrystals in the air and using pre‐synthesized precursor stock solutions. More importantly, we find that the reaction yields mainly nanocubes of different sizes (bulk‐like and quantum confined) in the temperature range of 175 to 100 °C, however, further decreasing the reaction temperature leads to the formation of NPls. Surprisingly, higher research temperature results in hexapod NCs with distinct optical properties compared to nanocubes due to their size differences. The nanocrystals synthesized by this approach are as monodisperse as those prepared by classical hot‐injection synthesis under an inert atmosphere, with a standard deviation ranging from 10 to 15 %. Despite the synthesis in the air, the NCs exhibit narrow emission without applying any size‐selective separation process. Besides, the halide composition, and thus the PL emission, could be precisely tuned by using different ratios of corresponding PbX_2_ (X=Cl, Br, or I) precursor solutions. Interestingly, we find that the PbX_2_ precursor's ratio (PbBr_2_/PbCl_2_ and PbI_2_/PbBr_2_) has a near‐linear relationship with the PL peak position of the mixed halide perovskite nanocubes, this enables the find an exact ratio to obtain NCs with a specific PL emission. Importantly, the versatility of the synthetic approach is demonstrated by applying it to less‐explored FAPbBr_3_ NCs. We demonstrate the size and shape tunability from bulk‐like 3D FAPbBr_3_ nanocubes to strongly quantum‐confined 0D FAPbBr_3_ nanocubes with 3D‐quantum confinement and 2D nanoplatelets with 1D‐quantum confinement by the reaction temperature. Interestingly, only below 50 °C the reaction yields FAPbBr_3_ nanoplatelets. We strongly believe that this user‐friendly synthetic approach presenting here will not only be useful for the large‐scale synthesis of lead halide perovskite NCs for device applications but also could be routinely used for the synthesis of various other perovskites NCs and their derivatives.

## Conflict of interest

The authors declare no conflict of interest.

## Supporting information

As a service to our authors and readers, this journal provides supporting information supplied by the authors. Such materials are peer reviewed and may be re‐organized for online delivery, but are not copy‐edited or typeset. Technical support issues arising from supporting information (other than missing files) should be addressed to the authors.

Supporting Information
