# Supplementary material for: Dimensionality Control of Inorganic and Hybrid Perovskite Nanocrystals by Reaction Temperature: From No‐Confinement to 3D and 1D Quantum Confinement
Source: Angew Chem Int Ed Engl. 2021 Nov 15;60(51):26677–84. doi: 10.1002/anie.202109308 (PMC9299153; doi:10.1002/anie.202109308)
Supplement: Supplementary file 1 — Supporting Information [file ANIE-60-26677-s001.pdf]

Angewandte Chemie
Eine Zeitschrift der Gesellschaft Deutscher Chemiker

Supporting Information

# Dimensionality Control of Inorganic and Hybrid Perovskite Nanocrystals by Reaction Temperature: From No-Confinement to 3D and 1D Quantum Confinement

*Clara Otero-Martínez, Daniel García-Lojo, Isabel Pastoriza-Santos, Jorge Pérez-Juste, and Lakshminarayana Polavarapu**

anie_202109308_sm_miscellaneous_information.pdf

## Experimental Procedures

### Materials

Cesium carbonate ($Cs_2CO_3$, 99,9%), Coumarin-153 ($C_{16}H_{14}F_3NO_2$, 99%), formamidine acetate salt ($CH_4N_2{\cdot}C_2H_4O_2$, 99%), lead (II) chloride ($PbCl_2$, 98%), lead (II) bromide ($PbBr_2$, >98%), lead (II) iodide ($PbI_2$, 99%), 1-octadecene (ODE, $C_{18}H_{36}$, 90%) oleic acid (OA, $C_{18}H_{34}O_2$, 90%), and oleylamine (OLA, $C_{18}H_{37}N$, 70%) were purchased from Merck. Hexane ($C_6H_{14}$, 95%) was supplied by Alfa Aesar and Acetone ($C_3H_6O$, 99.9%) by VWR Chemicals. All chemicals were used without further purification.

**Preparation of a stock of cesium-oleate (Cs-OL).** In a typical synthesis, 407 mg of $Cs_2CO_3$ (1.25 mmol) and 1.25 mL of oleic acid (3.5 mmol) were added to 20 mL of 1-octadecene in a 50 ml sample vial. The resulting mixture was heated at 150 ºC under stirring until the salt is completely dissolved. The thus prepared Cs-oleate (Cs-OL) solution remains stable for over 6 months. The Cs-oleate complex generally precipitates at room temperature, however, it can be easily dissolved again by continuous stirring at 120 ºC.

**Preparation of a stock of formamidinium-oleate (FA-OL).** In a typical synthesis, 521 mg of formamidine acetate (5 mmol) was mixed with 20 mL of oleic acid (56 mmol). The resulting mixture was heated at 150 ºC under stirring until the salt is completely dissolved.

**Preparation of a stock of different lead halides ($PbX_2$)**. In a typical synthesis, the lead halide salt (267 mg of $PbCl_2$ (0.94 mmol), 345 mg of PbBr2 (0.94 mmol) or 433 mg of PbI2 (0.94 mmol)) was added to a mixture of 2.5 mL of OLA (5.3 mmol), 2.5 mL of oleic acid (7.0 mmol) and 25 mL of 1-octadecene in a 50 ml sample vial. The resulting mixture was heated at 150 ºC under continuous stirring until the salt completely dissolves.

**Synthesis of $CsPbCl_nBr_{3-n}$ and $CsPbBr_nI_{3-n}$ (n = 0-3) perovskite NCs**. In a typical synthesis, 6 mL of the mixed halide precursor solutions were prepared by mixing different volumes of the $PbX_2$ stock solutions depending on the desired halide ratio. The specific volumes employed to prepare mixed halide perovskite NCs are provided in Table 1. The resulting mixed halide solution in a 20 mL glass vial was heated on a hot-plate until the temperature of the precursor solution reaches 175 $^oC$, and then, 400 µL of the pre-heated Cs-OL stock solution was swiftly injected into it under vigorous stirring (1,000 rpm). After 5 s, the vial was removed from the hot-plate and placed in an ice-water bath to quench the reaction. Subsequently, the thus obtained colloidal dispersion was purified by centrifugation at 8000 rpm for 10 min. Then, the supernatant was discarded to remove the unreacted precursors and ligands and the pellet was redispersed in 5 mL of hexane. The colloidal dispersion was centrifuged again at 5,000 rpm for 10 min) to remove larger NCs in the sediment. The supernatant was characterized by absorption, PL, and TEM. The scalability of the synthesis was tested by increasing the precursor volumes 25 times while the other reaction conditions and purification process remains unchanged. *Note*: a typical mercury thermometer was used to monitor the temperature up to 175 $^oC$, while metal tip was used to probe the temperature higher than 175 $^oC$.

**Table 1.** Specific volumes of each $PbX_2$ precursor employed to prepare *$CsPbCl_nBr_{3-n}$ and $CsPbBr_nI_{3-n}$ (n = 0-3)*, their emission wavelengths, and size of the obtained NCs.

| Theoretical Composition* | V $PbCl_2$ precursor (mL) | V $PbBr_2$ precursor (mL) | V $PbI_2$ precursor (mL) | Emission wavelength (nm) | Size of the NCs (nm) |
|---|---|---|---|---|---|
| $CsPbCl_3$ | 6 | 0 | 0 | 402 | 5.3 ± 1.2 |
| $CsPbCl_2Br_1$ | 4 | 2 | 0 | 433 | 5.9 ± 0.7 |
| $CsPbCl_{1.5}Br_{1.5}$ | 3 | 3 | 0 | 455 | 6.7 ± 1.0 |
| $CsPbCl_1Br_2$ | 2 | 4 | 0 | 484 | 7.1 ± 0.9 |
| $CsPbBr_3$ | 0 | 6 | 0 | 514 | 8.3 ± 1.0 |
| $CsPbBr_2I_1$ | 0 | 4 | 2 | 546 | 8.5 ± 0.9 |
| $CsPbBr_{1.5}I_{1.5}$ | 0 | 3 | 3 | 602 | 9.0 ± 0.9 |
| $CsPbBrI_2$ | 0 | 2 | 4 | 656 | 9.8 ± 0.8 |
| $CsPbI_3$ | 0 | 0 | 6 | 686 | 11.1 ± 1.4 |

** This nomenclature is based on the quantity of each precursor added but not refers to the final composition. other reaction conditions and the purification process remains unchanged. Note: a typical mercury thermometer monitored the temperature of the reaction medium.*

**Synthesis of $CsPbBr_3$ or $CsPbI_3$ with tunable dimensions by temperature control.** The synthesis is the same as it is described above for $CsPbX_3$ NCs, however, the reaction was performed under different temperatures. In a typical synthesis, 6 mL of the $PbBr_2$ or $PbI_2$ stock solution in a 20 mL glass sample vial was heated to the desired temperature (50 to 175 $^oC$) and then 400 µL of the pre-heated Cs-OL stock solution was quickly added into it under vigorous stirring (1,000 rpm). After 5 s, the vial was removed from the hot-plate and placed it in an ice-water bath to quench the reaction. The purification process was the same as described above for $CsPbBr_3$ NCs. However, it was important to precipitate the $CsPbBr_3$ NCs synthesized at reaction temperatures lower than 150 $^oC$ by adding 14 mL of acetone to the as-prepared colloidal dispersion. The resultant colloidal dispersion was purified by centrifugation at 8000 rpm for 10 min and then the sediment was dispersed in 5 mL of hexane. Finally, the colloidal dispersion was further centrifuged at 2000 rpm for 10 min to remove larger particles in the sediment.

*Note*: a typical mercury thermometer monitored the temperature of the reaction medium.

**Synthesis of $FAPbBr_3$ at different reaction temperatures.** In a typical synthesis, 6 mL of the $PbBr_2$ stock solution in a 20 mL glass vial was heated on a hotplate to different temperatures before introducing FA-oleate. After reaching the desired reaction temperature (25 to 175 ºC), 2,5 mL of the pre-heated FA-OL stock solution was swiftly introduced into the reaction medium under vigorous stirring (1,000 rpm). After 5 s, the vial was removed from the hot-plate and placed it in an ice-water bath to quench the reaction. The colloidal dispersions were purified in a similar way as it is described for $CsPbBr_3$ NCs. However, in the case of colloidal dispersions obtained at 40 to 80 $^oC$ reaction temperatures, 14 mL of acetone was added to precipitate the NCs before applying centrifugation step. This enabled the isolation of small nanocubes from the nanoplatelets present in the colloidal dispersion. Interestingly, the colloidal NCs

obtained at 25 $^{o}$C mainly contain nanoplatelets, while the NCs synthesized at above 100 $^{o}$C mainly nanocubes.

*Note*: the temperature of the reaction medium was monitored by a typical mercury thermometer.

**Characterization**

UV-Vis extinction spectra were carried out using a Cary 8454 spectrophotometer (Agilent). Photoluminescence spectra of all the colloidal solutions were obtained with a FluoroMax-3 (Horiba Jobin Yvon) at an excitation wavelength of 350 nm. Quartz cuvettes with an optical path length of 1 cm were used for both optical analyses. PLQY measurements were performed through a standard procedure previously reported[1] using Coumarin-153 as a reference dye. Transmission electron microscopy (TEM) images were obtained with a JEOL JEM 1010 transmission electron microscope operating at an acceleration voltage of 100 kV. Scanning transmission electron microscopy (STEM) images were acquired using a JEOL JEM-2010F operating at an acceleration voltage of 200 kV. XRD patterns were obtained using a **PANalytical -XPERT-PRO X-Ray** diffractometer, equipped with a 1.54 kW Cu Kα, operating at 40 kV and 30 mA. The samples for the XRD measurements were prepared by drop-casting a concentrated NC solution into a silicon wafer.

**Table S2.** Summary of the optical properties (photoluminescence (PL) and photoluminescence quantum yield (PLQY)), shape and the dimensions of the $CsPbBr_3$ and $FAPbBr_3$ NCs prepared under different reaction temperatures.

| Composition | Reaction temperature (ºC) | Shape | Size / thickness (nm) | PL (nm) | PLQY (%) |
|---|---|---|---|---|---|
| $CsPbBr_3$ | 50 | Nanoplatelets | 2.0 ± 0.4 | 455 | 5 |
| $CsPbBr_3$ | 80 | Nanoplatelets + Nanocubes | 1.9 ± 0.6 (NPls)<br>3.2 ± 0.8 (NCs) | 455- 479 | 96 |
| $CsPbBr_3$ | 100 | Nanoplatelets + Nanocubes | 1.9 ± 0.4 (NPls)<br>3.7 ± 0.6 (NCs) | 455 -488 | 93 |
| $CsPbBr_3$ | 125 | Nanocubes | 5.7 ± 0.9 | 511 | 96 |
| $CsPbBr_3$ | 150 | Nanocubes | 7.9 ± 0.4 | 515 | 96 |
| $CsPbBr_3$ | 175 | Nanocubes | 9.3 ± 1.1 | 515 | 95 |
| $CsPbBr_3$ | 215 | Hexapods | 20 ± 1.0 | 520 | 5 |
| $FAPbBr_3$ | 25 | Nanoplatelets | 1.7 ± 0.3 | 435 | 25 |
| $FAPbBr_3$ | 40 | Nanocubes | 2.5 ± 0.6 | 476 | 45 |
| $FAPbBr_3$ | 60 | Nanocubes | 4.1 ± 0.8 | 482 | 43 |
| $FAPbBr_3$ | 80 | Nanocubes | 5.1 ± 1.0 | 498 | 55 |
| $FAPbBr_3$ | 130 | Nanocubes | 6.4 ± 0.9 | 510 | 96 |
| $FAPbBr_3$ | 175 | Nanocubes | 8.1 ± 1.0 | 527 | 94 |
| $FAPbBr_3$ | 200 | Nanocubes | 14.5 ± 1.5 | 534 | 94 |

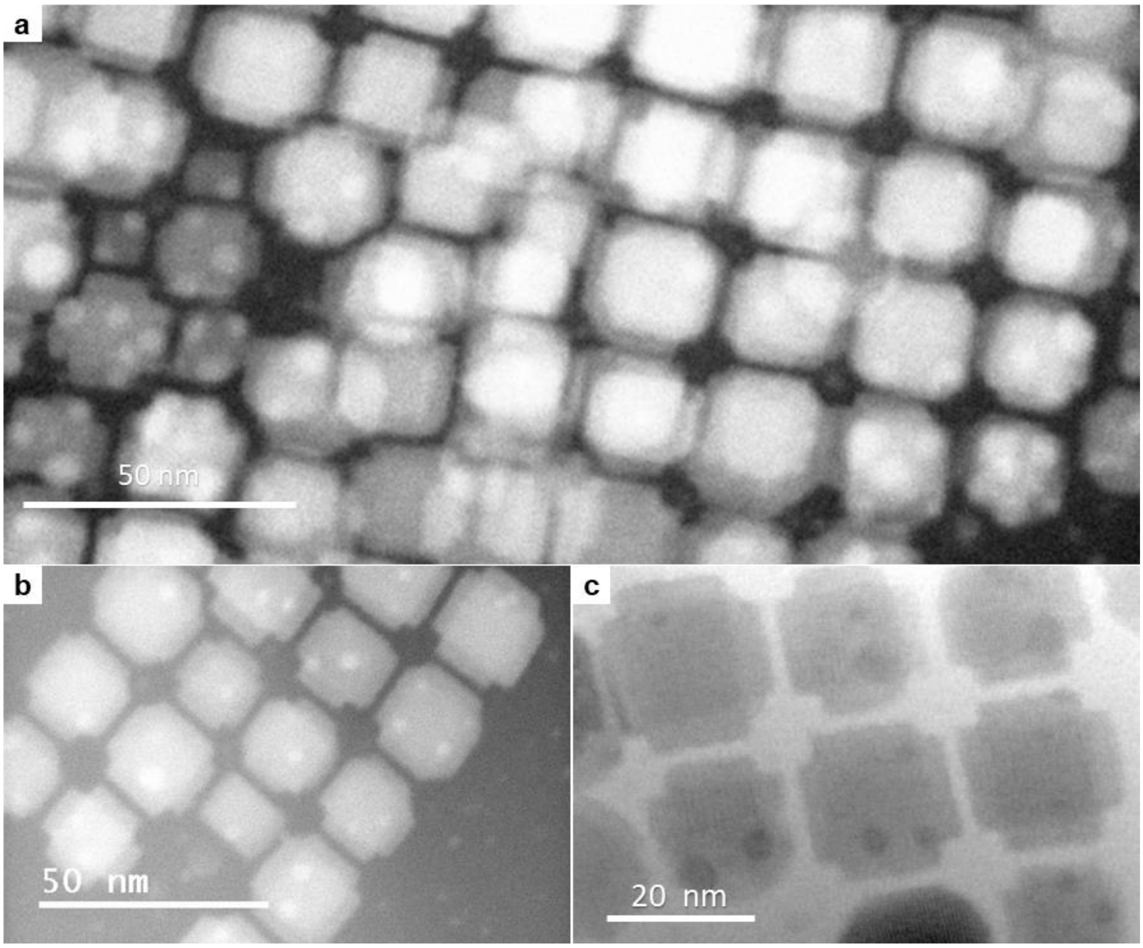


**Figure S1.** a-c) Representative STEM images of $CsPbBr_3$ nanoparticles synthesized at 215 $^{o}C$ at different magnifications.

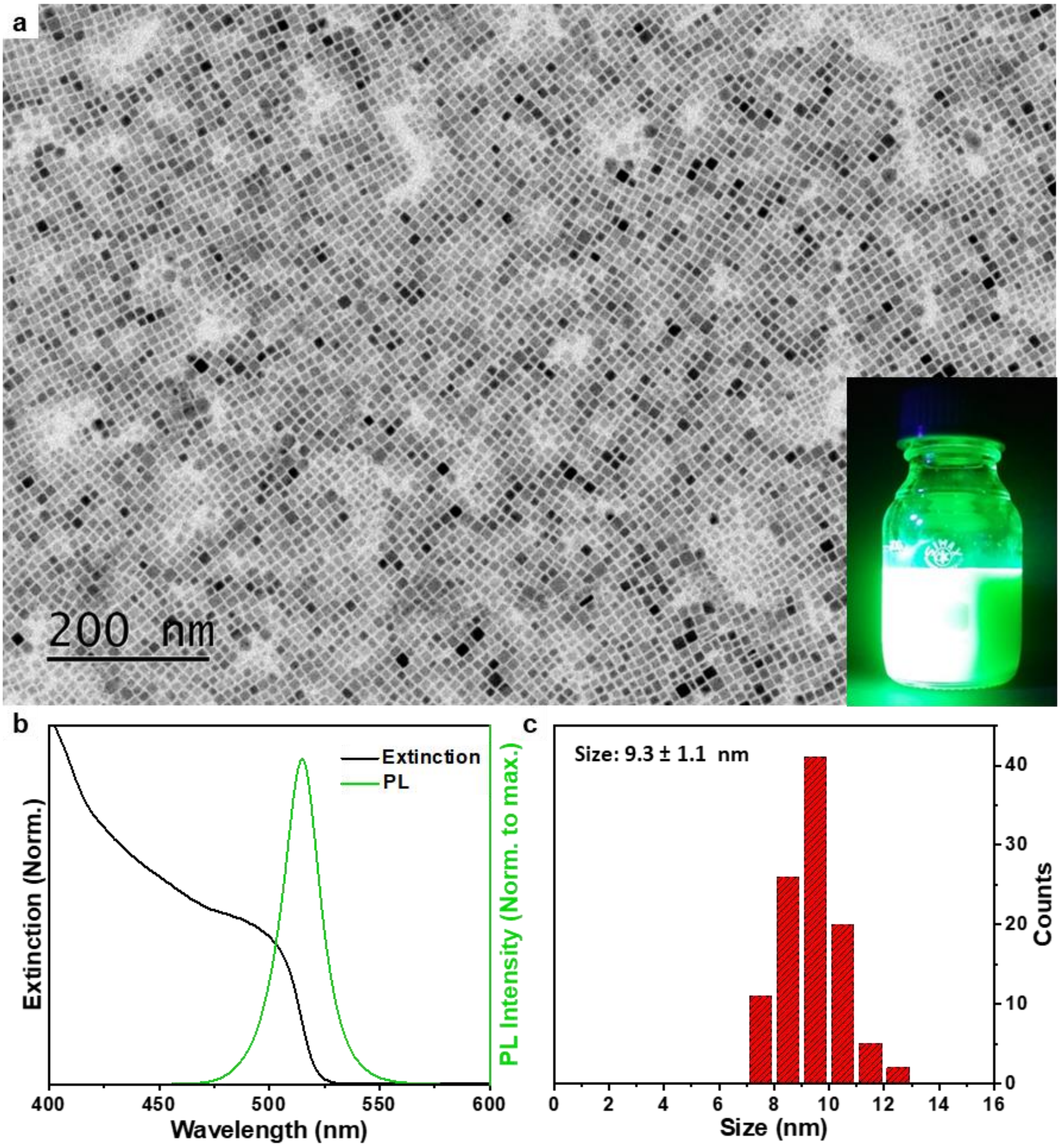


**Figure S2.** a) Representative TEM image of the $CsPbBr_3$ cubic nanocrystals obtained by scale-up the reaction 25 times. b) UV-Vis extinction (black line) and PL (green line) spectra of the colloidal dispersion of $CsPbBr_3$ nanocrystals in hexane. c) Histogram obtained from the analysis of TEM images. The average size of the perovskite nanocrystals is 9.3 ± 1.1 nm.

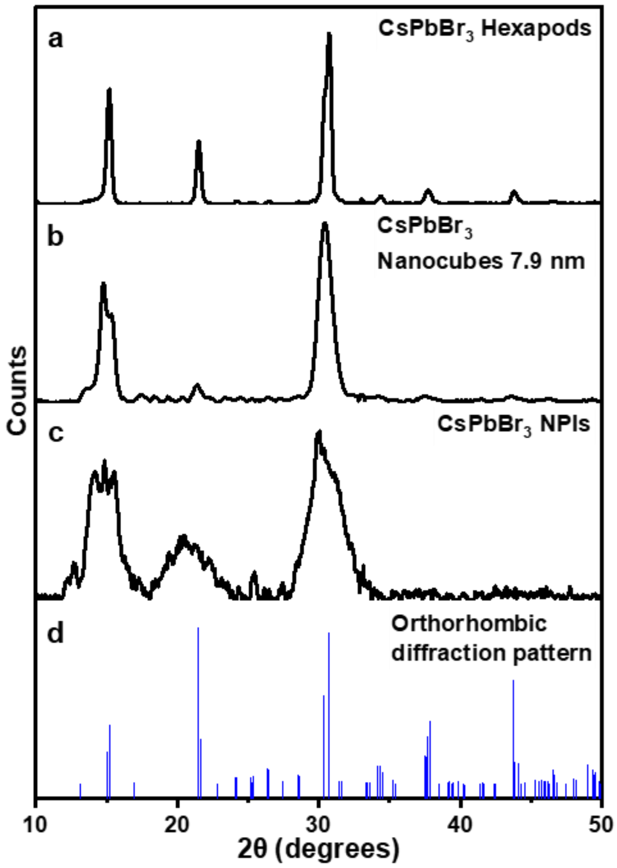


**Figure S3.** XRD pattern of $CsPbBr_3$ hexapods (a), nanocubes of 7.9 nm (b), 3ML NPls (c), and reference orthorhombic phase (obtained from the cif file of Ref.[2])(d).

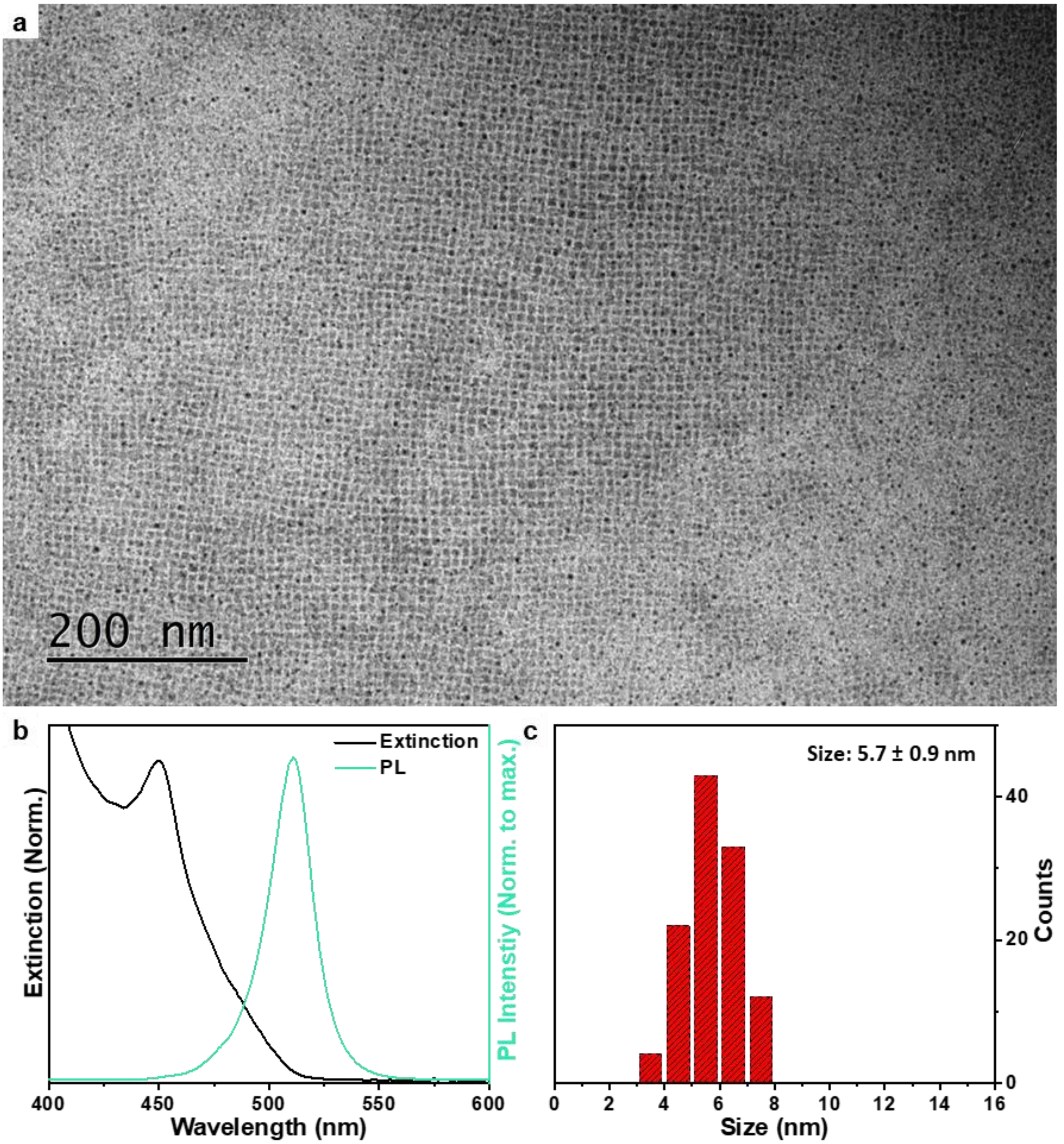


**Figure S4**. a) Representative TEM image of $CsPbBr_3$ nanocubes obtained at 125 °C. **b)** UV-Vis extinction (black line) and PL (green line) spectra of the colloidal $CsPbBr_3$ nanocrystals in hexane. **c)** Histogram obtained from the analysis of TEM images. The average size of the perovskite nanocrystals is 5.7 ± 0.9 nm. The higher energy absorption peak at ~450 nm suggest that presence of NPls in the colloidal solution, however the corresponding the PL peak intensity is very low due to their low PLQY.

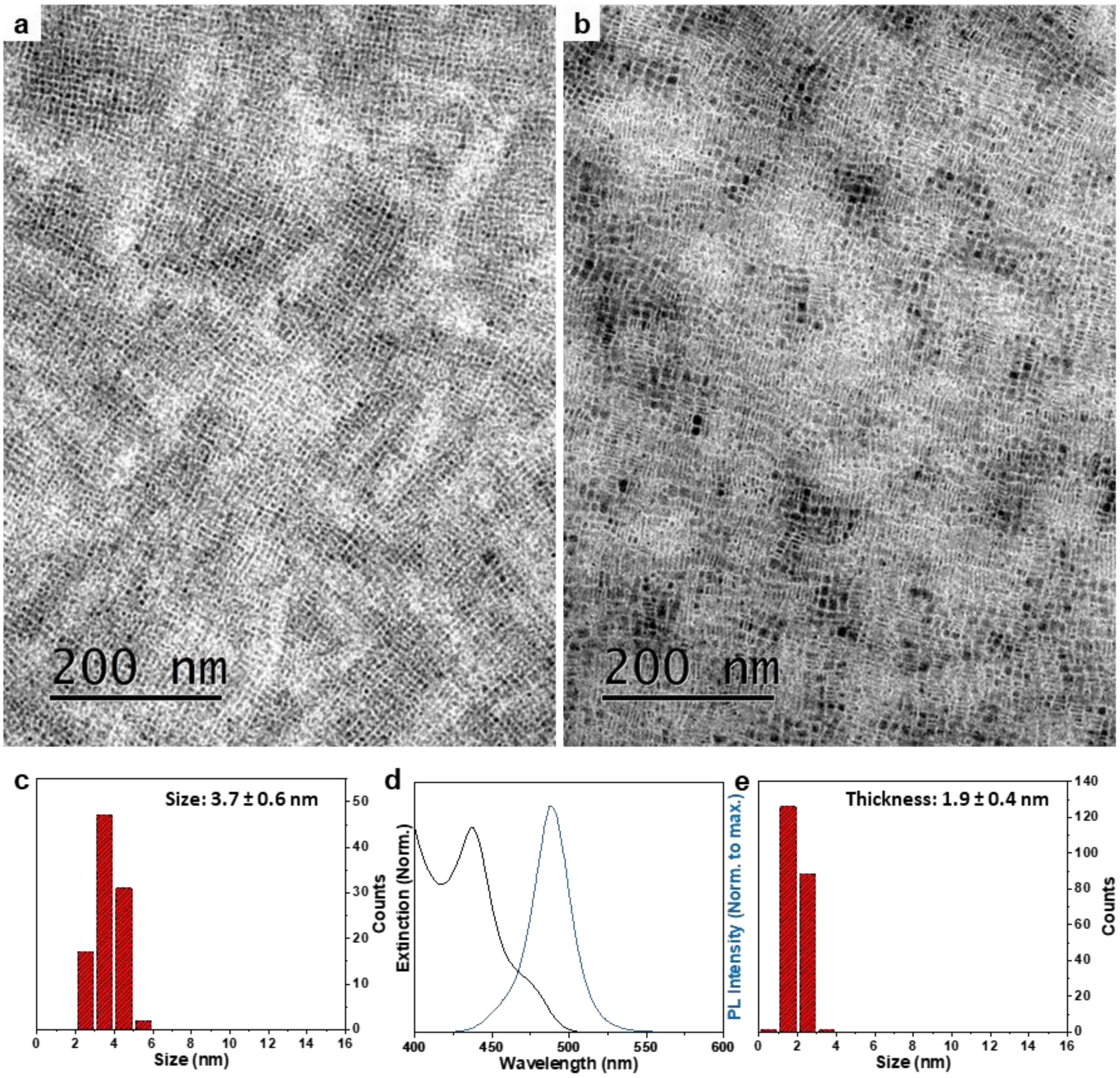


**Figure S5**. (a-b) Representative TEM image of nanocubes (a) and nanoplatelets (b) present in the colloidal solution of $CsPbBr_3$ NCs obtained at 100 $^oC$. The images suggest that there is a segregation of NPls and nanocubes in different areas of the TEM grid. The histograms obtained from the analysis of TEM images are provided in the insets. The average size of the perovskite nanocubes is 3.7 ± 0.6 nm, while the average thickness of the perovskite nanoplatelets is 1.9 ± 0.4 nm. (c) UV-Vis extinction (black line) and PL (blue line) spectra of the corresponding colloidal solution of $CsPbBr_3$ nanocrystals in hexane. The absorption peak at higher energy corresponds to the 3- monolayer think (1.9 ± 0.4 nm) NPls and the lower energy corresponds to the 3.7 ± 0.6 nm nanocubes. The PL spectra show a small shoulder peak at higher energy and it corresponds to weakly emissive NPls.

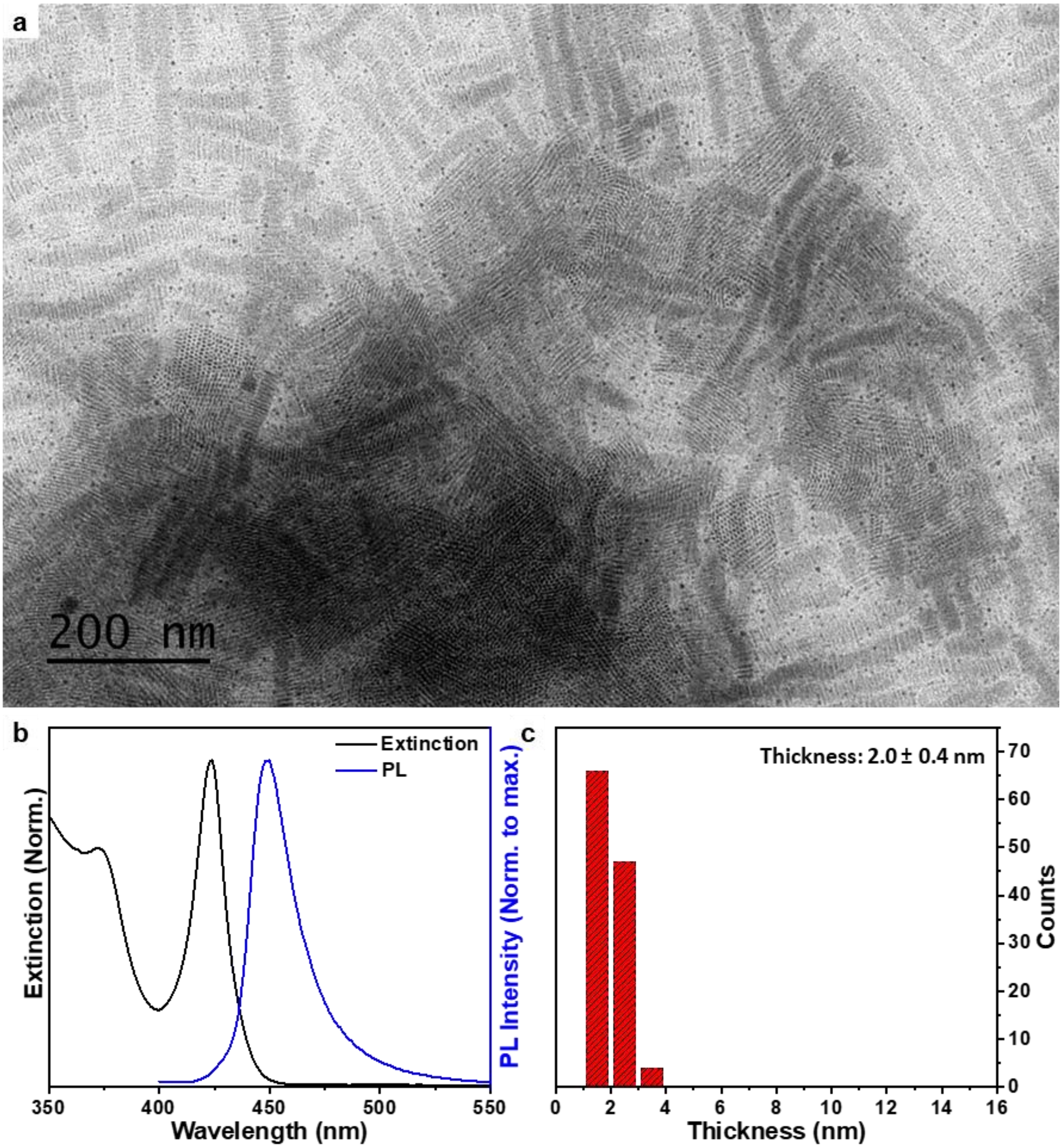


**Figure S6**. a) Representative TEM image of $CsPbBr_3$ nanoplatelets obtained at 50 $^{o}$C. b) UV-Vis extinction (black line) and PL (blue line) spectra of the corresponding $CsPbBr_3$ nanoplatelets in hexane. c) Histogram obtained from the analysis of TEM images. The average thickness of the perovskite nanoplatelets is 2.0 ± 0.4 nm.

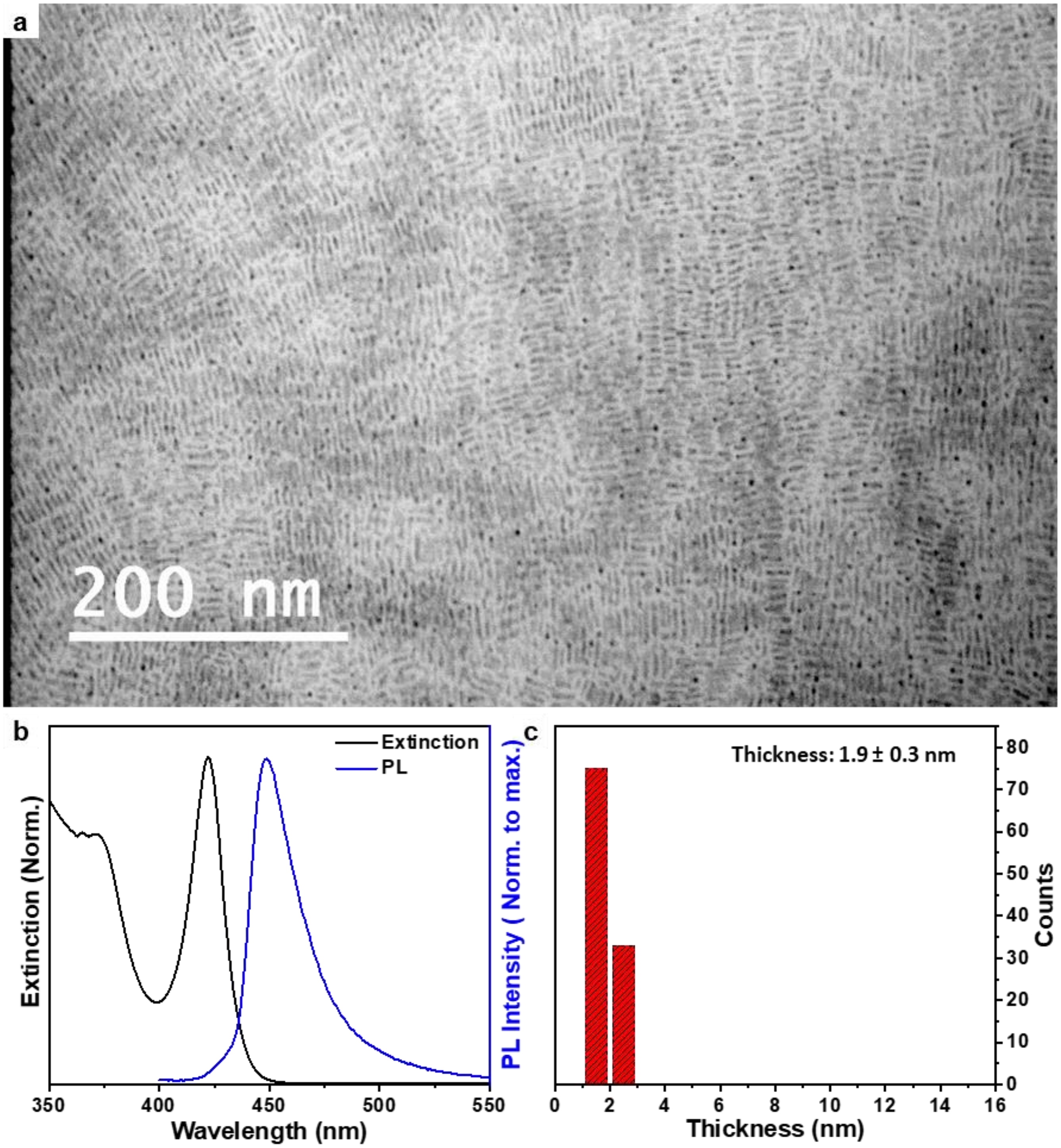


**Figure S7**. a) Representative TEM image of the $CsPbBr_3$ nanoplatelets obtained by up-scaling the synthesis 20 times. b) UV-Vis extinction (black line) and PL (blue line) spectra of the corresponding $CsPbBr_3$ nanoplatelets in hexane. c) Histogram obtained from the analysis of TEM images. The average thickness of the perovskite nanoplatelets is 1.9 ± 0.3 nm.

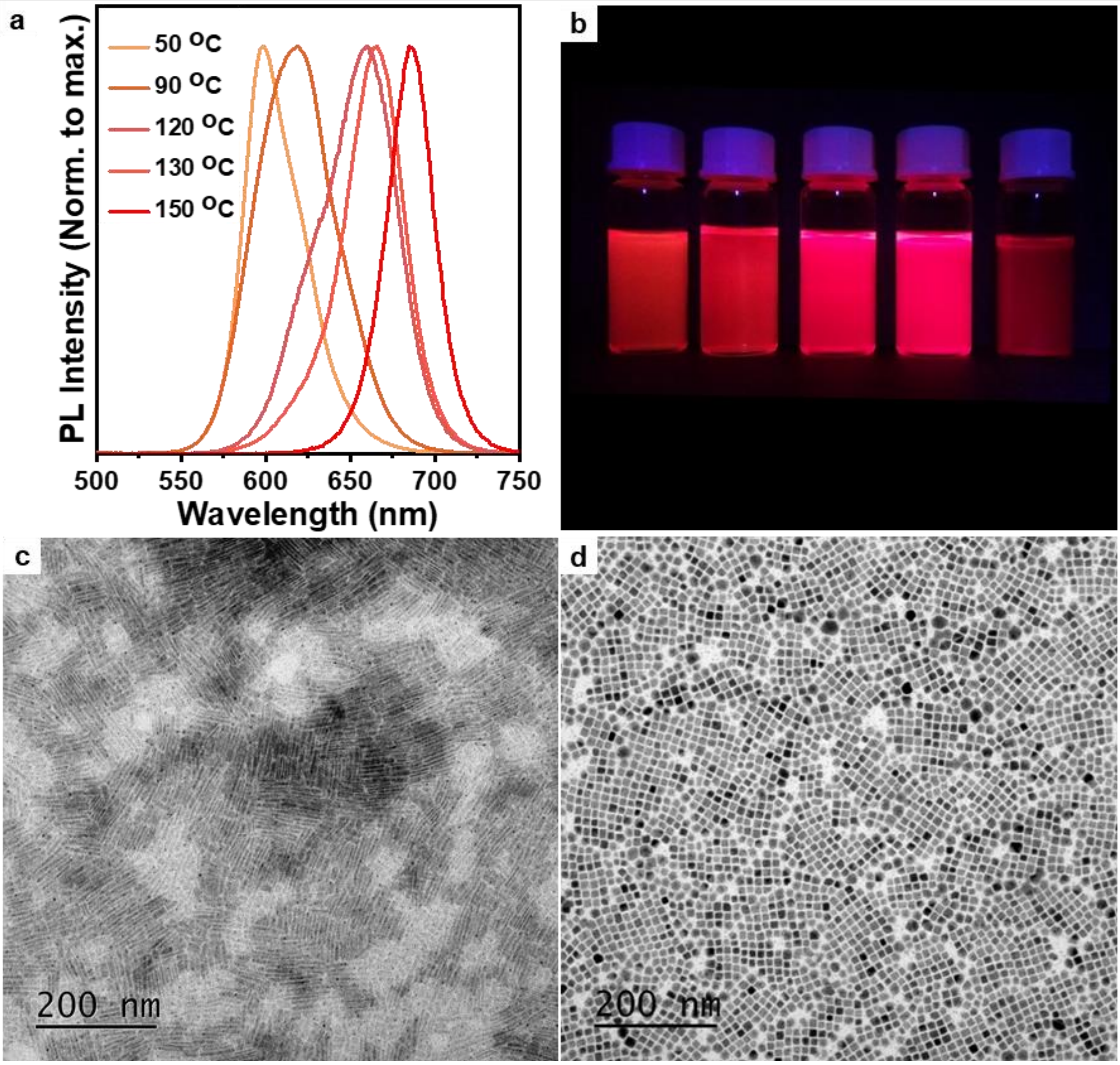


**Figure S8**. a) Normalized photoluminescent spectra obtained for the $CsPbI_3$ perovskite NCs synthesized at different reaction temperatures. b) Photograph of the corresponding $CsPbI_3$ colloidal dispersions under UV light. c-d) Representative TEM image of $CsPbI_3$ nanoplatelets (c)and nanocubes obtained at 50 $^oC$ and 150 $^oC$, respectively.

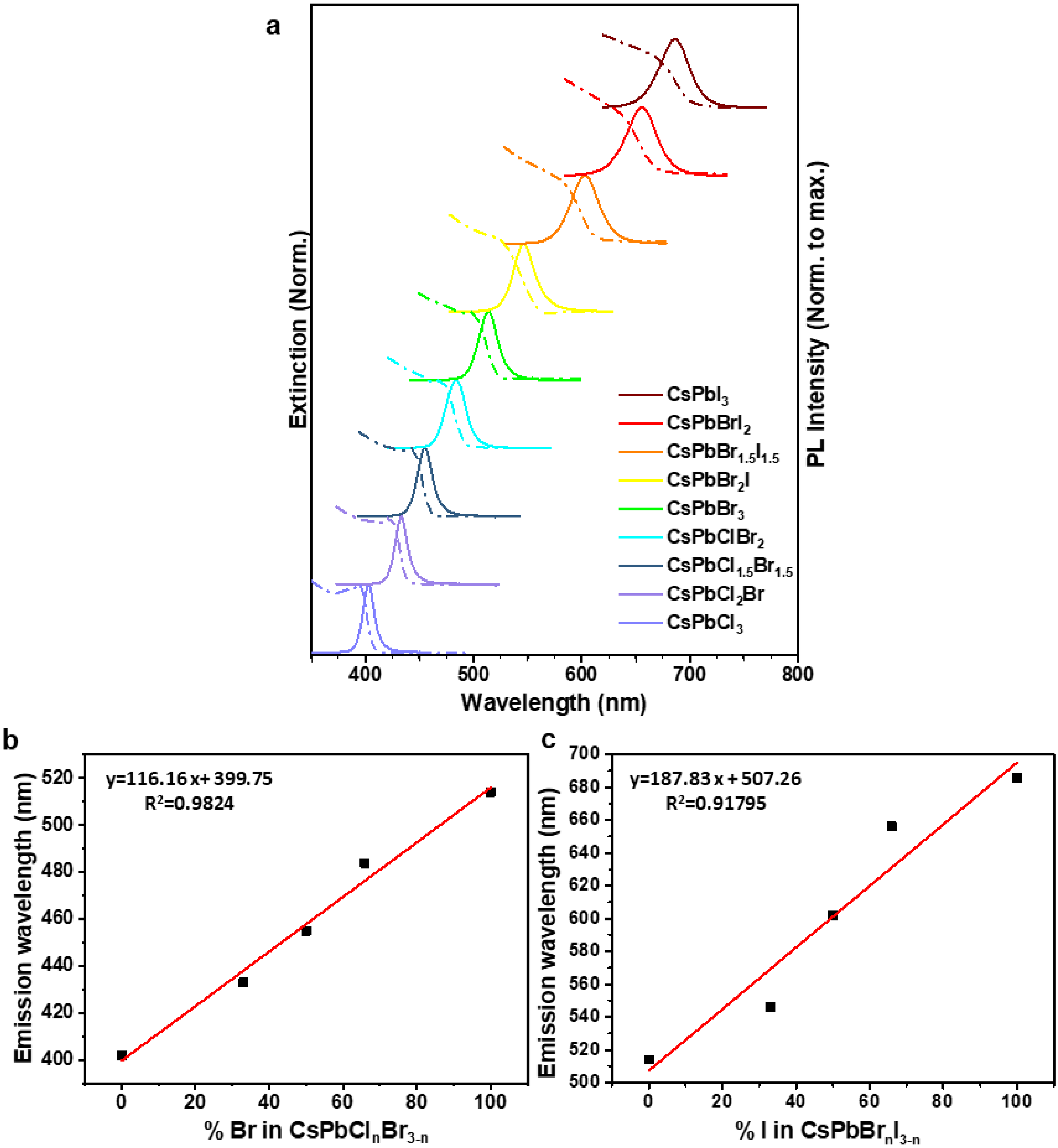


**Figure S9**. a) UV-Vis extinction and PL spectra of cesium lead mixed halide perovskites nanocrystals in hexane. The chemical formulation is based on the halide precursor concentration. b-c) Emission wavelength of mixed halide perovskite NCs as a function of different ratios of halide precursors employed for the synthesis of $CsPbCl_nBr_{3-n}$ (b) and $CsPbBr_nI_{3}$-n (c).

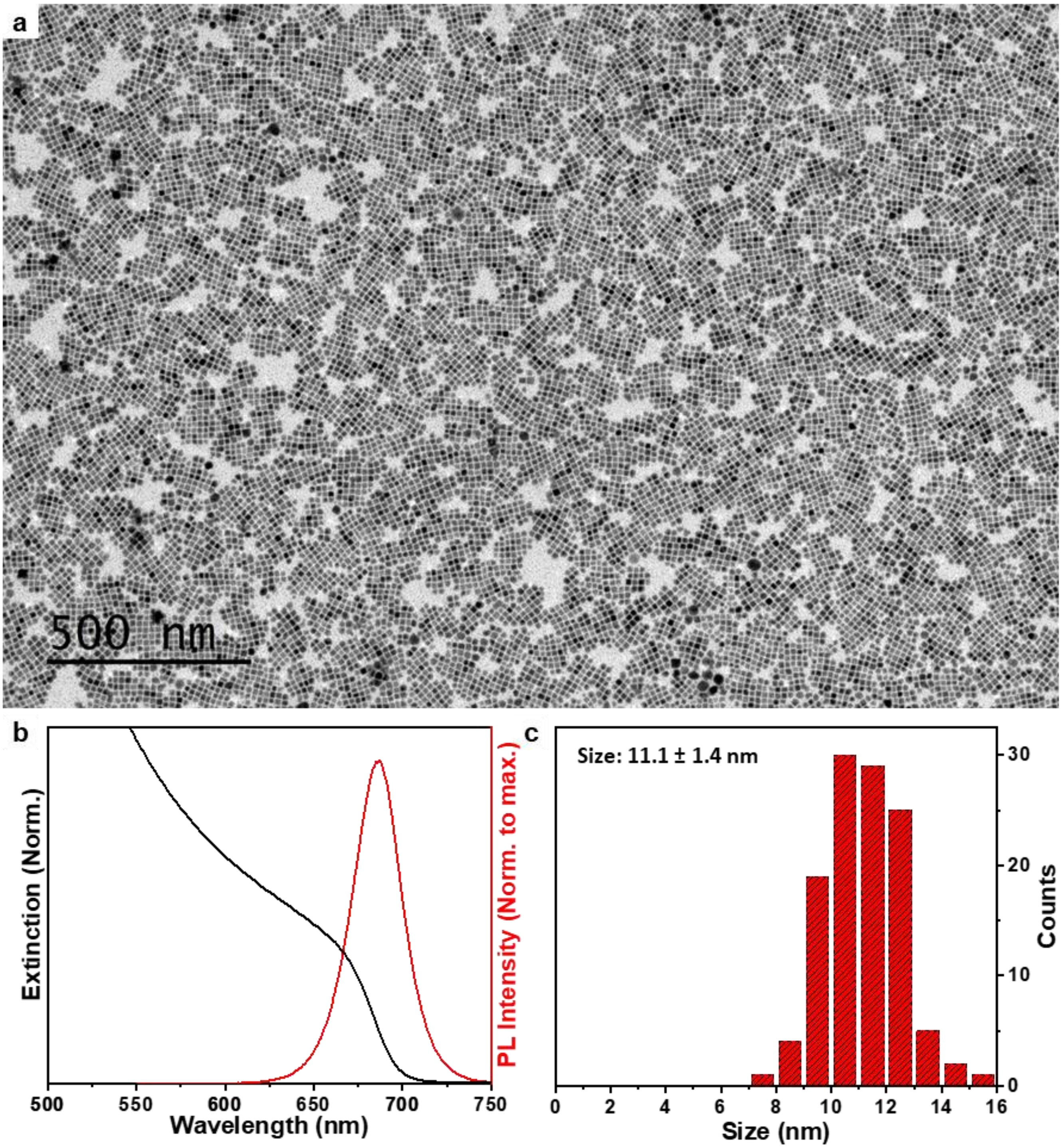


**Figure S10**. a) Representative TEM image of $CsPbI_3$ cubic nanocrystals synthesized at 175 ºC. b) UV-Vis extinction (black line) and PL (red line) spectra of the colloidal $CsPbI_3$ NCs in hexane. c) Histogram obtained from the analysis of TEM images. The average size of the perovskite nanocrystals is 11.1 ± 1.4 nm.

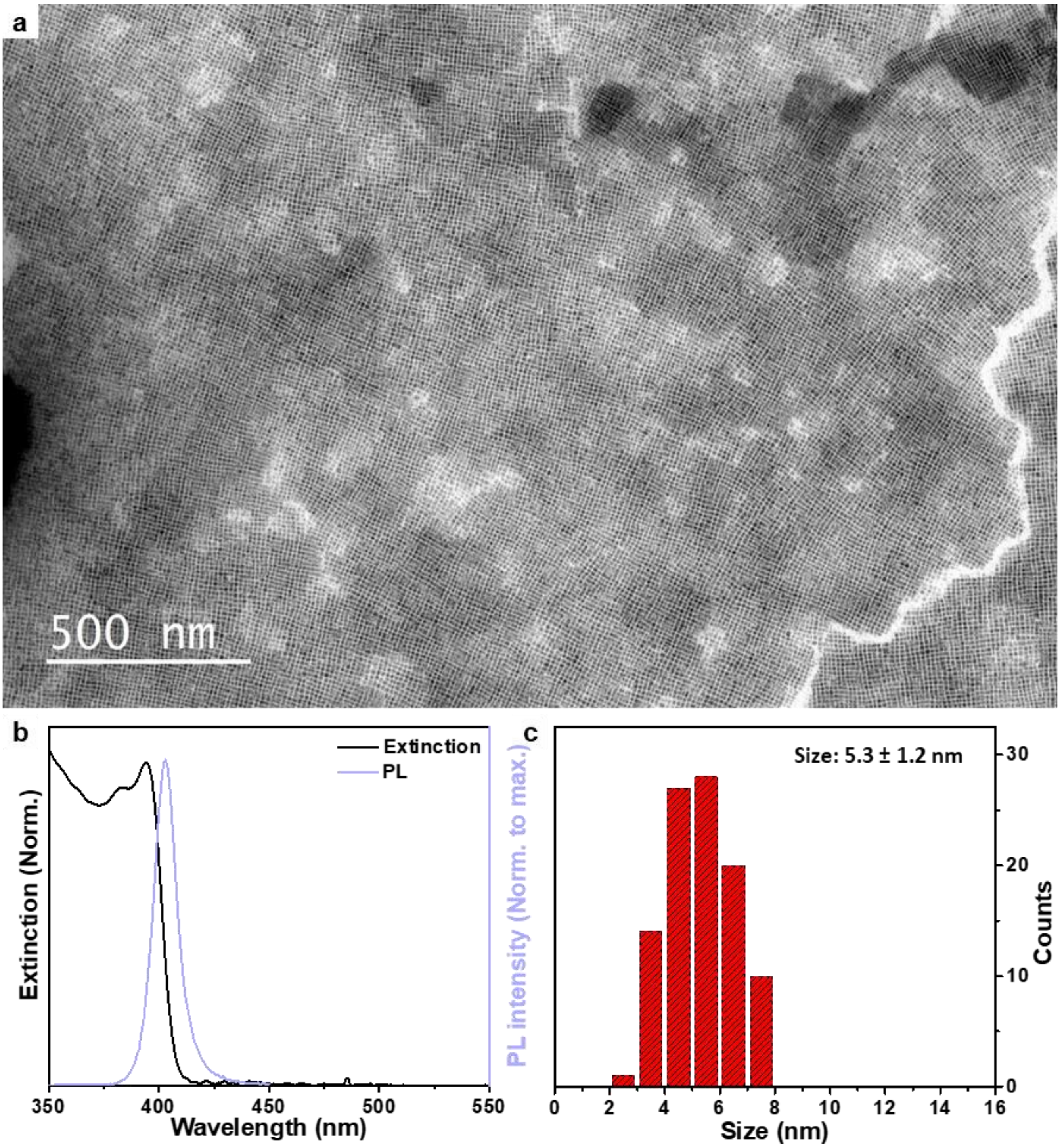


**Figure S11**. a) Representative TEM image of $CsPbCl_3$ cubic nanocrystals synthesized at 175 ºC. b) UV-Vis extinction (black line) and PL (red line) spectra of the colloidal $CsPbCl_3$ NCs in hexane. c) Histogram obtained from the analysis of TEM images. The average size of the perovskite nanocrystals is 5.3 ± 1.2 nm.

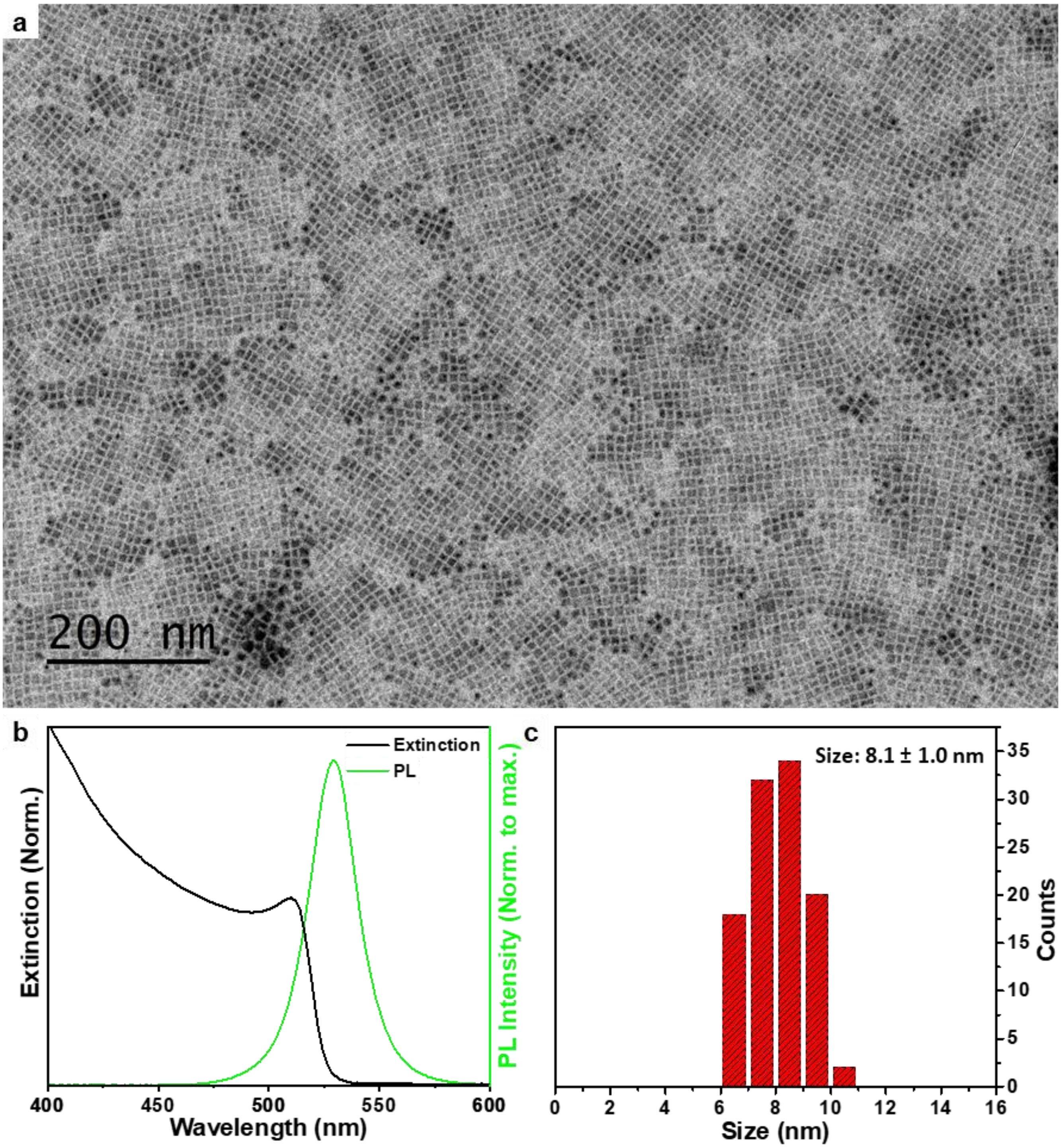


**Figure S12**. a) Representative TEM image of $FAPbBr_3$ nanocubes obtained at 175 $^{o}$C. b) UV-Vis extinction (black line) and PL (green line) spectra of the corresponding colloidal dispersion of nanocubes in hexane. c) Histogram obtained from the analysis of TEM images. The average thickness of the perovskite nanocrystals is 8.1 ± 1.0 nm.

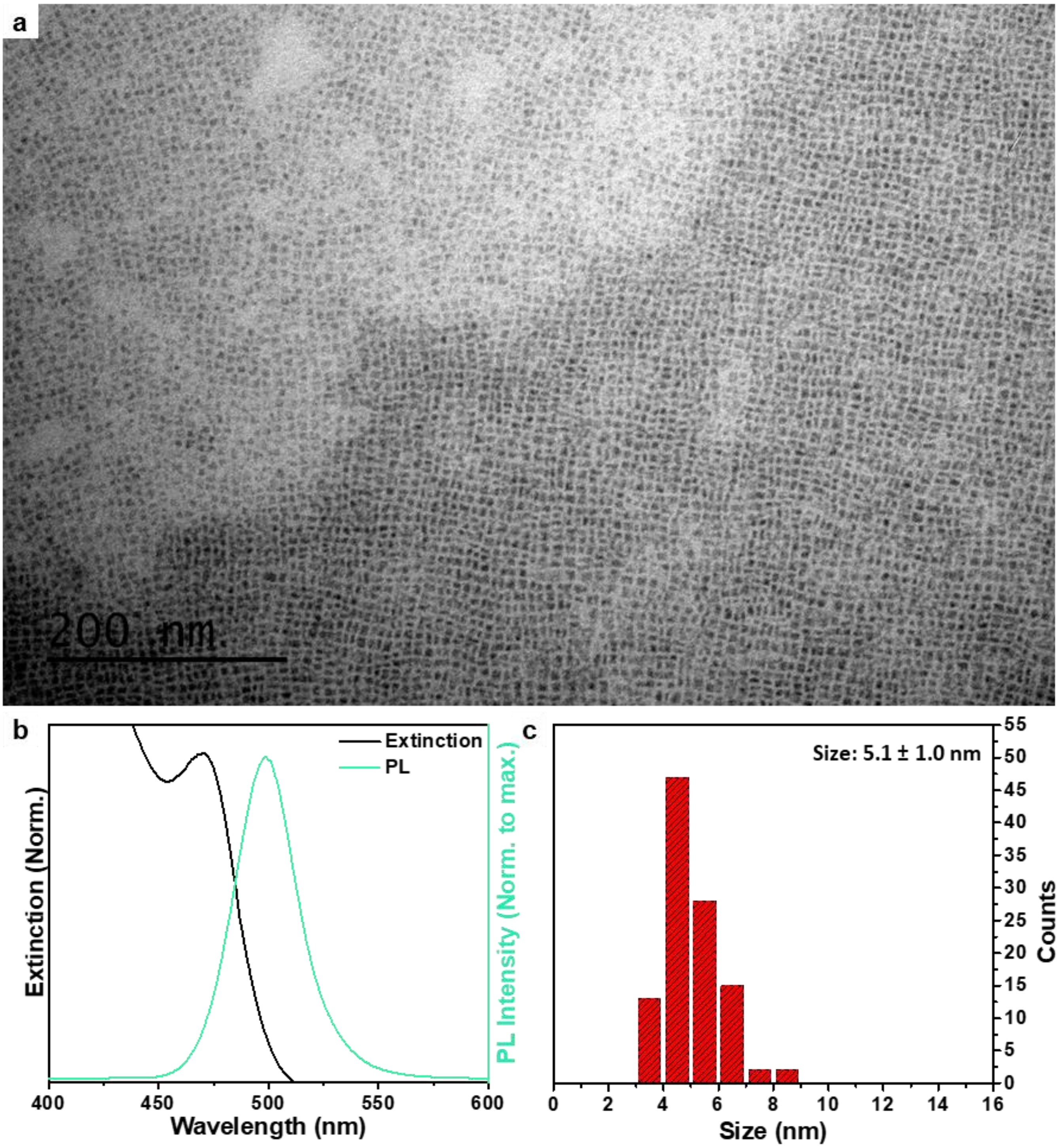


**Figure S13**. a) Representative TEM image of $FAPbBr_3$ nanocubes obtained at 80 ºC. b) UV-Vis extinction spectrum (black line) and PL (green line) spectra of the corresponding colloidal dispersion of $FAPbBr_3$ nanocubes in hexane. c) Histogram obtained from the analysis of TEM images. The average size of the perovskite nanocrystals is 5.1 ± 1.0 nm.

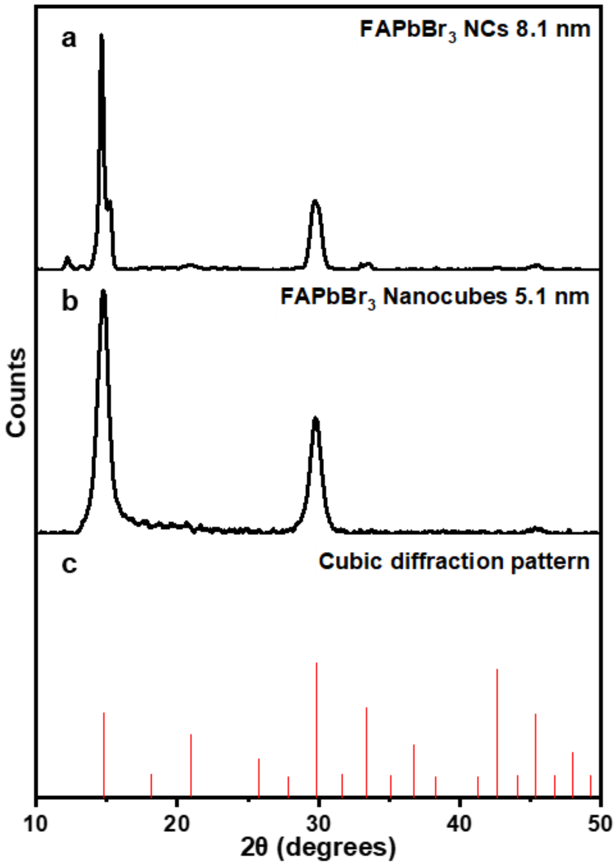


**Figure S14.** a-b) XRD pattern of $FAPbBr_3$ nanocubes of sizes 8.1 ± 1.0 nm (a) 5.1 ± 1.0 nm (b), and reference cubic phase (obtained from the cif file of Ref.[3])(c). It is worth mentioning that it was difficult to obtain XRD patterns of $FAPbBr_3$ NPls as they tend to degrade when subjected to washing using methyl acetate or acetone.

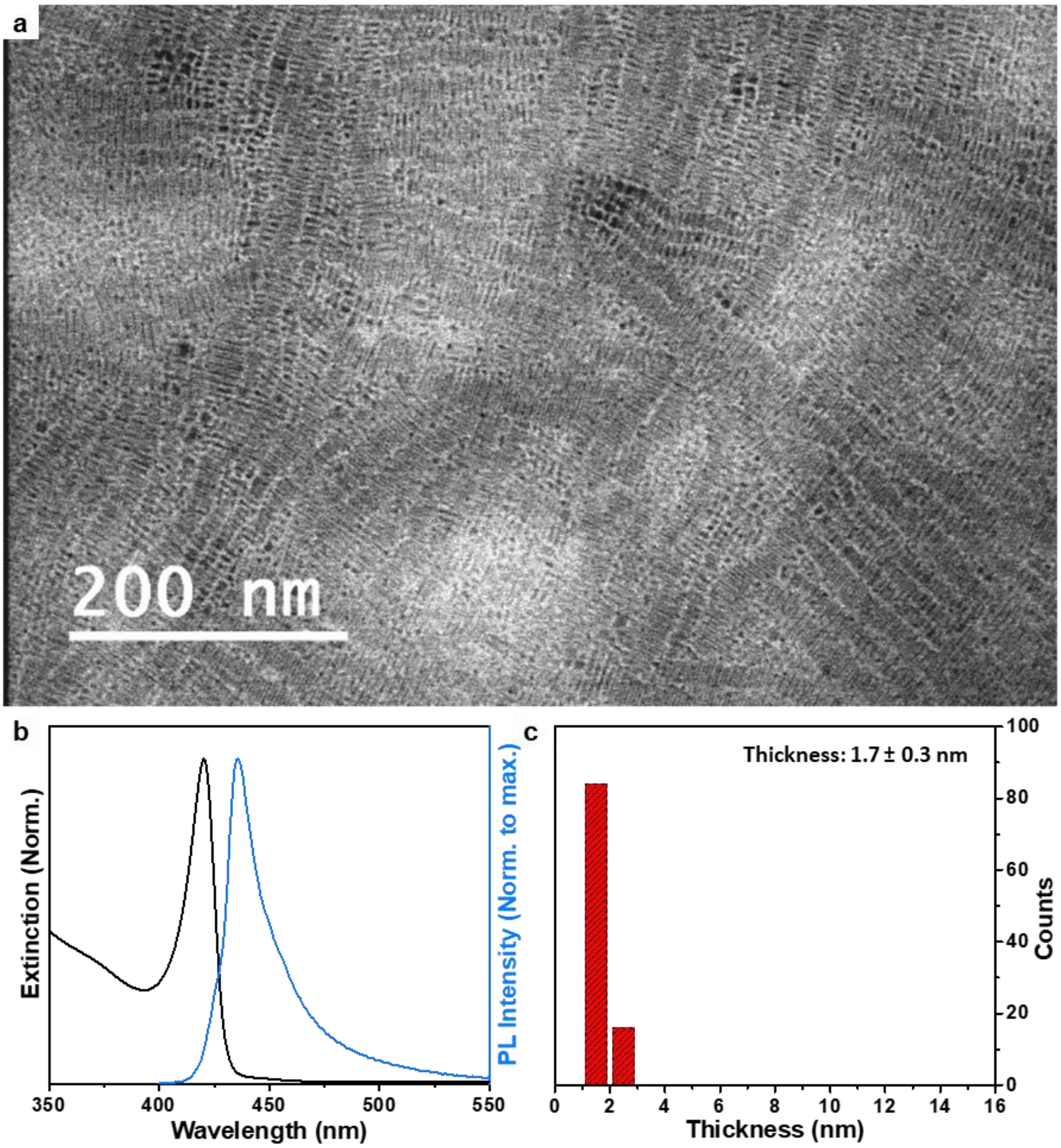


**Figure S15**. a) Representative TEM image of $FAPbBr_3$ nanoplatelets obtained at 25 $^oC$. b) UV-Vis extinction spectrum (black line) and PL (blue line) spectra of the corresponding colloidal dispersion of $FAPbBr_3$ nanoplatelets in hexane. c) Histogram obtained from the analysis of TEM images. The average thickness of the perovskite nanoplatelets is 1.7 ± 0.3 nm.

**References**

[1] M. Grabolle, M. Spieles, V. Lesnyak, N. Gaponik, A. Eychmüller, U. Resch-Genger, *Analytical Chemistry* **2009**, *81*, 6285-6294.
[2] M. Rodová, J. Brožek, K. Knížek, K. Nitsch, *J. Therm. Anal. Calorim.* **2003**, *71*, 667-673.
[3] L. Leppert, S. E. Reyes-Lillo, J. B. Neaton, *J. Phys. Chem. Lett.* **2016**, *7*, 3683-3689.
